# Supplementary material for: A host–guest approach to combining enzymatic and artificial catalysis for catalyzing biomimetic monooxygenation
Source: Nat Commun. 2020 Jun 9;11:2903. doi: 10.1038/s41467-020-16714-7 (PMC7283336; doi:10.1038/s41467-020-16714-7)
Supplement: Supplementary file 1 — Supplementary Information [file 41467_2020_16714_MOESM1_ESM.pdf]

< Supplementary information >

A host-guest approach to combining enzymatic and artificial catalysis for catalyzing biomimetic monooxygenation

Zhao et al.

Supplementary Methods

Experimental materials

All chemicals were of reagent grade quality obtained from commercial sources. Unless stated otherwise, all the chemicals and solvents were used without further purification. ^{1}H NMR and ^{13}C NMR spectra were measured on a Bruker 400 M spectrometer. ESI mass spectra were carried out on an HPLC-Q-Tof mass spectrometer using methanol as mobile phase. UV-Vis spectra were measured on a HP 8453 spectrometer. The elemental analyses of C, H and N were performed on a Vario EL III elemental analyzer. The solution fluorescent spectra were measured on Edinburgh FS-920. Isothermal Titration Calorimetry (ITC) was performed on a Nano ITC (TA Instruments Inc. – Waters LLC).

All electrochemical measurements were carried out under argon gas at room temperature performed on a ZAHNER ENNIUM electrochemical workstation with a conventional three-electrode system with a homemade Ag/AgCl electrode as a reference electrode, a platinum silk with 0.5 mM diameter as a counter electrode, and glassy carbon electrode as a working electrode.

The oxidation of reaction was made in a 15 mL flask. The flask was sealed with a septum. The reaction vial was full with O_2 by bubbling oxygen for 20 min under atmospheric pressure at room temperature. The oxygen in reaction vials was adequate with an oxygen balloon. The reaction vials were used a water filter to absorb heat to maintain a constant temperature and the vials were irradiated with a 3 W fluorescent lamp while stirring with a magnetic stirring bar.

Preparation and Characterizations

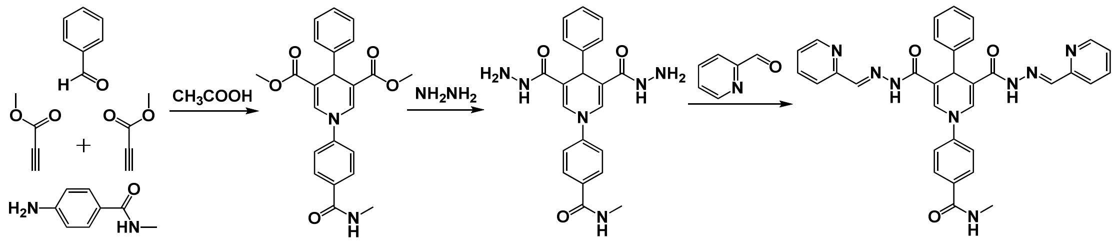

Compound 1 Compound 2 H2ZPA

Supplementary Figure 1. The synthetic route of the H_2**ZPA**.

Synthesis of compound **1**

Methyl propiolate (1.68 g, 20.0 mmol), benzaldehyde (1.06 g, 10.0 mmol), and 4-amino-*N*-methylbenzamide (1.50 g, 10.0 mmol) in glacial acetic acid (2.0 mL) were heated at 80°C for 30 min[1]. After cooling, the mixture was poured into water (20 mL) and stirred for 1 h. The solid product was filtered and washed with Et_2O (10 mL × 3) to give pure product, which was recrystallized by ethanol. Yield: 2.41 g, 59.3%. ^{1}H NMR ($CDCl_3$, 400 MHz, ppm): δ 7.89 (d, 2H), 7.74 (s, 2H), 7.37 (d, 4H), 7.31-7.27 (m, 1H), 7.20 (t, 1H), 6.21 (d, 1H), 4.99 (s, 1H), 3.69 (s, 6H), 3.07 (d, 3H).

Synthesis of compound **2**

A mixture solution of 80% hydrazine hydrate (50 mL) and compound **1** (4.06 g, 10.0 mmol) was stirred at 85°C for 12 h. The yellow precipitate was formed, which was collected by filtration, washed with ethanol and dried in vacuum. Yield: 1.93 g, 47.5%. ^{1}H NMR (400 MHz, DMSO-*d*$_6$, ppm): δ 9.17 (s, 2H), 8.35 (q, 1H), 7.60-7.52 (m, 5H), 7.24-7.18 (m, 4H), 7.12-7.08 (m, 1H), 6.58-6.50 (m, 1H), 5.13 (s, 1H), 4.17 (s, 4H), 2.79 (s, 3H).

Synthesis of ligand H_2**ZPA**

Compound **2** (4.06 g, 10.0 mmol) was added to an ethanol solution (50 mL) containing 2-pyridylaldehyde (2.35 g, 22.0 mmol). After 5 drops of acetic acid was added, the mixture was heated at 85°C for 12 h according to the reference[2]. The yellow solid was collected by filtration, washed with methanol and dried in vacuum. Yield: 4.10 g, 71.4%. ^{1}H NMR (400 MHz, DMSO-*d*$_6$, ppm): δ 11.61 (s, 2H), 8.59 (d, J = 4.8 Hz, 2H), 8.54 (q, J = 4.4 Hz, 1H), 8.26 (s, 2H), 8.02 (d, J = 8.8 Hz, 2H), 7.90-7.83 (m, 6H), 7.72 (d, J = 8.8 Hz, 2H), 7.41-7.26 (m, 6H), 7.18-7.14 (m, 1H), 5.35 (s, 1H), 2.82 (d, J = 4.4 Hz, 3H). ^{13}C NMR ($CDCl_3$, 101 MHz, ppm): δ 165.5, 163.3, 153.3, 149.4, 146.0, 145.3, 144.5, 136.7, 131.2, 130.8, 128.6, 128.2, 127.8, 126.5, 124.1, 119.7, 119.1, 112.6, 56.0, 26.2. Elemental analysis calcd for $C_{33}H_{28}N_8O_3 \cdot H_2O$: H, 5.02; C, 65.77; N,

18.59%. Found: H, 5.10; C, 66.27; N, 18.57%. ESI-MS calcd for $C_{33}H_{28}N_8O_3$ 584.23, found 585.24 $[M+H]^+$, 607.22 $[M+Na]^+$.

Preparation of Zn−**ZPA**

$Zn(BF_4)_2{\cdot}H_2O$ (25.7 mg, 0.10 mmol) and H_2**ZPA** (58.4 mg, 0.10 mmol) were dissolved in CH_3CH_2OH/CH_3CN (v/v: 1/1) to give a yellow solution and keep stirring overnight. The X-ray quality yellow block crystals were grown by diffusing diethyl ether into the complex's solution. Yield: 60%. ^{1}H NMR (400 MHz, DMSO-d_6, ppm): 11.78 (s, 2H), 8.56-8.48 (m, 3H), 8.27 (s, 2H), 8.01 (d, J = 8.4 Hz, 2H), 7.89 (s, 6H), 7.70 (d, J = 8.4 Hz, 2H), 7.42-7.24 (m, 6H), 7.18-7.14 (t, J = 7.2 Hz, 1H), 5.33 (s, 1H), 2.80 (d, J = 4.0 Hz, 3H). Elemental analysis calcd for $Zn_4C_{132}H_{110}N_{32}O_{12}B_6F_{24}{\cdot}CH_3CN$: H, 4.07; C, 57.57; N, 3.51%. Found: H, 4.01; C, 57.73; N, 3.53%. ESI-MS: m/z: 864.5278 $[H_3Zn_4(\mathbf{ZPA})_4]^{3+}$.

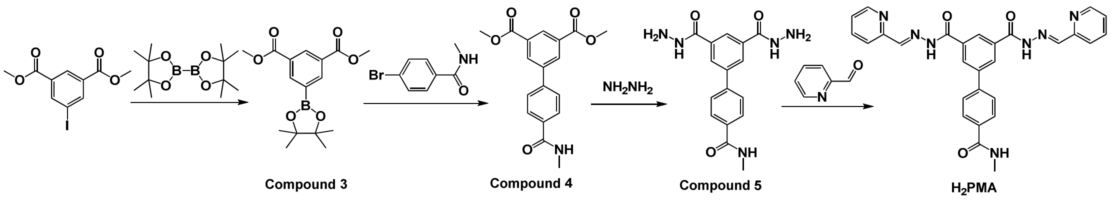

Supplementary Figure 2. The synthetic route of the H_2**PMA**.

Synthesis of compound **3**

Dimethyl 5-iodoisophthalate (18.72 g, 58.5 mmol), bis(pinacolato)diboron (14.86 g, 58.5 mmol), Pd(acetate)$_2$ (0.36 g, 1.6 mmol) and potassium acetate (15.70 g, 0.16 mol) were mixed in a 250 mL flask[3]. The flask was pumped under vacuum and refilled with N_2 three times, and then degrassed dry *N,N*-dimethylformamide (130 mL) was added. The mixture was stirred and heated to 90ºC for 12 h. The mixture cooled to room temperature and then 600 mL of water was added. The mixture was stirred vigorously for 10 min and the precipitates came out. The filtered precipitates were dissolved in CH_2Cl_2 and the organic solvent was removed under vacuum. The residue was purified by column chromatography on silica gel with 20% (ethylacetate/ Petroleum ether) to yield a white product (12.6 g, yield: 67.3%). ^{1}H NMR (400 MHz, $CDCl_3$, ppm): δ 8.76 (t, 1H), 8.64 (d, 2H), 3.95 (s, 6H), 1.36 (s, 12H).

Synthesis of compound **4**

Compound **3** (1.60 g, 5.0 mmol), 4-bromo-*N*-methylbenzamide (1.18 g, 5.5 mmol), CsF (1.52 g, 10.0 mmol) and tetrakis(triphenylphosphine)palladium(0) (0.15 g, 1.3 mmol) were mixed in a 100 mL flask connected to a condenser. The flask was degassed

under vacuum for 2 h, and then 50 mL of degassed dimethoxyenthane were added to the flask. The reaction mixture was heated to reflux under nitrogen atmosphere for 48 h. After removal of the organic solvent under vacuum, the residue was washed with water and extracted with chloroform three times. Combined organic layers were dried over $MgSO_4$ and filtered. The solvent was removed and the crude product purified by column chromatography on silica gel with 20% (ethyl acetate/petroleum ether) to yield a white product (1.12 g, yield: 68.4%). ^{1}H NMR (400 MHz, $CDCl_3$, ppm): δ 8.68 (t, 1H), 8.47 (s, 2H), 7.88 (d, 2H), 7.71 (d, 2H), 6.26 (s, 2H), 3.99 (s, 6H), 3.06 (d, 3H).

Synthesis of compound **5**

A mixture solution of 80% hydrazine hydrate (50 mL) and compound **4** (3.27 g, 10.0 mmol) was stirred at 85ºC for 12 h. The white precipitate was formed, which was collected by filtration, washed with ethanol and dried in vacuum. Yield: 2.00 g, 61.2%. ^{1}H NMR (400 MHz, DMSO-d_6, ppm): δ 9.98 (s, 2H), 8.52 (q, 1H), 8.30-8.28 (m, 3H), 7.98 (d, 2H), 7.91 (d, 2H), 4.58 (s, 4H), 2.82 (s, 3H).

Synthesis of ligand H_2**PMA**

Compound **5** (3.27 g, 10.0 mmol) was added to an ethanol solution (50 mL) containing 2-pyridylaldehyde (2.35 g, 22.0 mmol). After 5 drops of acetic acid was added, the mixture was heated at 85ºC under magnetic stirring for 12 h according to the reference[2]. The yellow solid was collected by filtration, washed with methanol and dried in vacuum. Yield: 4.10 g, 81.2%. ^{1}H NMR (400 MHz, DMSO-d_6, ppm): δ 12.30 (s, 2H), 8.65 (d, J = 4.4 Hz, 2H), 8.58-8.48 (m, 6H), 8.05-7.90 (m, 8H), 7.47-7.44 (m, 2H), 2.83 (d, J = 4.0 Hz, 3H). ^{13}C NMR ($CDCl_3$, 101 MHz, ppm): δ 166.0, 162.5, 153.1, 149.6, 148.6, 140.9, 139.8, 136.9, 134.4, 134.1, 129.1, 127.9, 127.0, 126.9, 124.5, 120.0, 26.3. Elemental analysis calcd for $C_{28}H_{23}N_7O_3$: H, 4.59; C, 66.52; N, 19.39%. Found: H, 4.69; C, 66.47; N, 19.27%. ESI-MS calcd for $C_{28}H_{23}N_7O_3$: 505.19, found 506.19 $[M+H]^+$, 528.18 $[M+Na]^+$.

Preparation of Zn−**PMA**

$Zn(BF_4)_2 \cdot H_2O$ (25.7 mg, 0.10 mmol) and H_2**PMA** (50.5 mg, 0.10 mmol) were dissolved in CH_3CH_2OH/CH_3CN (v/v: 1/1) to give a yellow solution. The X-ray quality yellow block crystals were grown by diffusing diethyl ether into the solution. Yield: 52%. ^{1}H NMR (400 MHz, DMSO-d_6, ppm): δ 12.32 (s, 2H), 8.65-8.48 (m, 8H), 8.05-7.93 (m, 8H), 7.47-7.45 (m, 2H), 2.83 (d, J = 4.0 Hz, 3H). Elemental analysis calcd for $Zn_4C_{112}H_{84}N_{28}O_{12} \cdot H_2O$: H, 3.78; C, 58.65; N, 17.10%. Found: H, 3.91; C, 58.85; N, 17.13%. ESI-MS: m/z: 759.1381 $[H_3Zn_4(\mathbf{PMA})_4]^{3+}$, 1138.1724 $[H_2Zn_4(\mathbf{PMA})_4]^{2+}$.

General procedure for the synthesis of the 3-substituted cyclobutanones

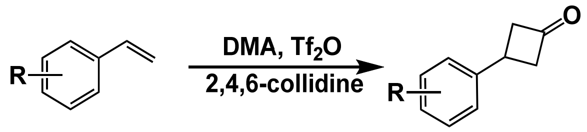

The products 3-substituted cyclobutanones were prepared according to the methods reported in the literature with a minor modification[4]. To a stirred solvent of DMA (1.19 mL, 12.8 mmol) in dichloroethane (13.0 mL) at −15ºC was added a solvent of Tf_2O (2.15 mL, 12.8 mmol) in dichloroethane (1.0 mL). The mixture was stirred at −15ºC for 30 minutes, then a solvent of styrene derivative (11.6 mmol) in dichloroethane (2.0 mL) was added, followed by a dropwise addition of a solvent of 2,4,6-collidine (1.69 mL, 12.8 mmol) in dichloroethane (1.0 mL). The mixture was heated to 130ºC for 6 h, and was allowed to cool to 80ºC. H_2O (34 mL) was added, and stirring was continued at 80ºC for 18 h. The mixture was allowed to cool to room temperature. The two layers were separated, and the organic layer was washed with water and brine. The organic layer was dried over Na_2SO_4, filtered, and the solvents were removed under reduced pressure. Purification by flash chromatography (EtOAc/heptane) yielded the target product.

Single Crystal X-ray Crystallography

The intensities of the Zn−**ZPA** and Zn−**PMA** were collected on a Bruker SMART APEX CCD diffractometer equipped with a graphite-monochromated Mo-Kα (λ = 0.71073 Å) radiation source; the data were acquired using the SMART and SAINT programs[5,6]. The structures were solved by direct methods and refined on F^2 by full-matrix least-squares methods with SHELXTL *version* 5.1 software[7]. In the structural refinement of Zn−**ZPA** and Zn−**PMA**, all the non-hydrogen atoms were refined anisotropically. Hydrogen atoms within the ligand backbones were fixed geometrically at calculated distances and allowed to ride on the parent non-hydrogen atoms. To assist the stability of refinements, one tetrafluoroborate anion and some benzene rings were restrained as idealized regular polygons and the thermal parameters on adjacent atoms in this anion and some benzene rings were restrained to be similar. The SQUEEZE subroutine in PLATON was used[8].

Crystal data of Zn−**ZPA**: $Zn_4C_{132}H_{110}N_{32}O_{12}\cdot 6BF_4\cdot 4CH_3CH_2OH\cdot 8H_2O$, M = 3447.26, Tetragonal, space group $P\bar{4}n2$, yellow block, a = 23.3087(12), b = 23.3087(12), c = 16.0801(16) Å, α = 90.00, β = 90.00, γ = 90.00, V = 8736.2(11) Å^3, Z = 2, Dc = 1.310 g cm^{-3}, μ(Mo-Kα) = 0.637 mm^{-1}, T = 180(2) K. 7675 unique reflections [R_{int} = 0.0783]. Final R_1 [with $I > 2\sigma(I)$] = 0.0860, wR_2 (all data) = 0.2230 for the data collected. CCDC number 1920570.

Crystal data of Zn−**PMA**: $Zn_4C_{112}H_{84}N_{28}O_{12}\cdot 6H_2O$, M = 2383.64, Tetragonal, space group $P\bar{4}$, yellow block, a = 16.4944(7), b = 16.4944(7), c = 14.3438(8) Å, α = 90.00, β = 90.00, γ = 90.00, V = 3902.4(4) Å^3, Z = 1, Dc = 1.014 g cm^{-3}, μ(Mo-Kα) = 0.664 mm^{-1}, T = 180(2) K. 9997 unique reflections [R_{int} = 0.1144]. Final R_1 [with $I > 2\sigma(I)$] = 0.0980, wR_2 (all data) = 0.2442 for the data collected. CCDC number 1920627.

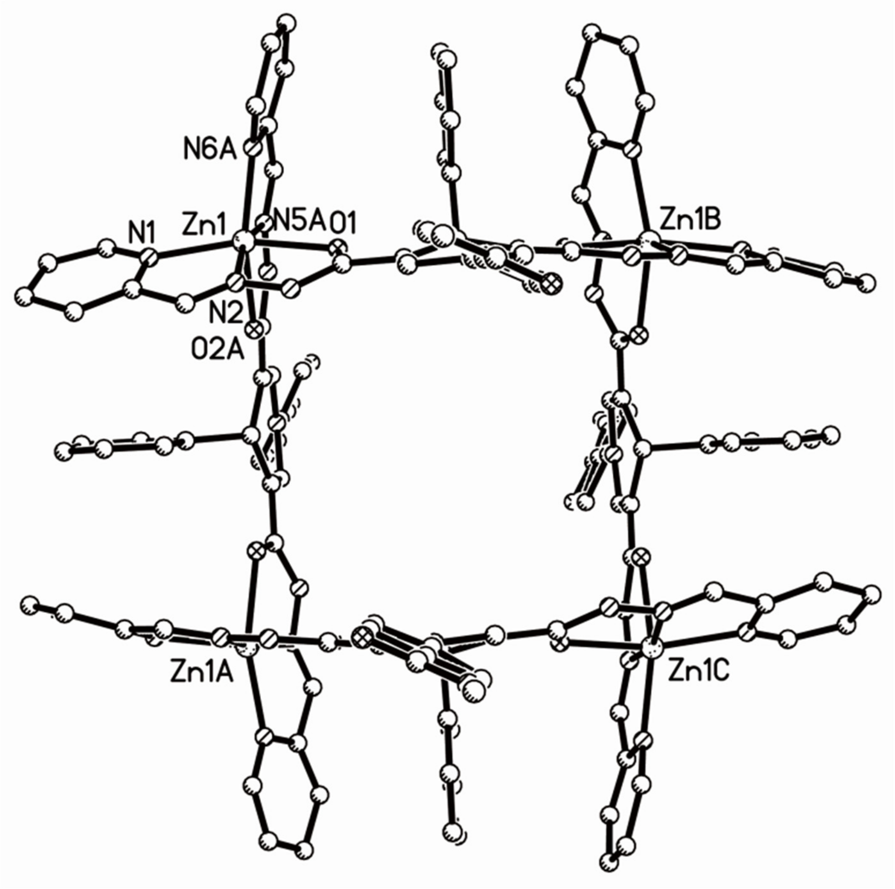

Supplementary Figure 3. A plot of the molecule Zn−**ZPA** is shown. Hydrogen atoms are omitted for clarity. Symmetry code A: -y+1, x, -z+1; B: y, -x+1, -z+1; C: -x+1, -y+1, z.

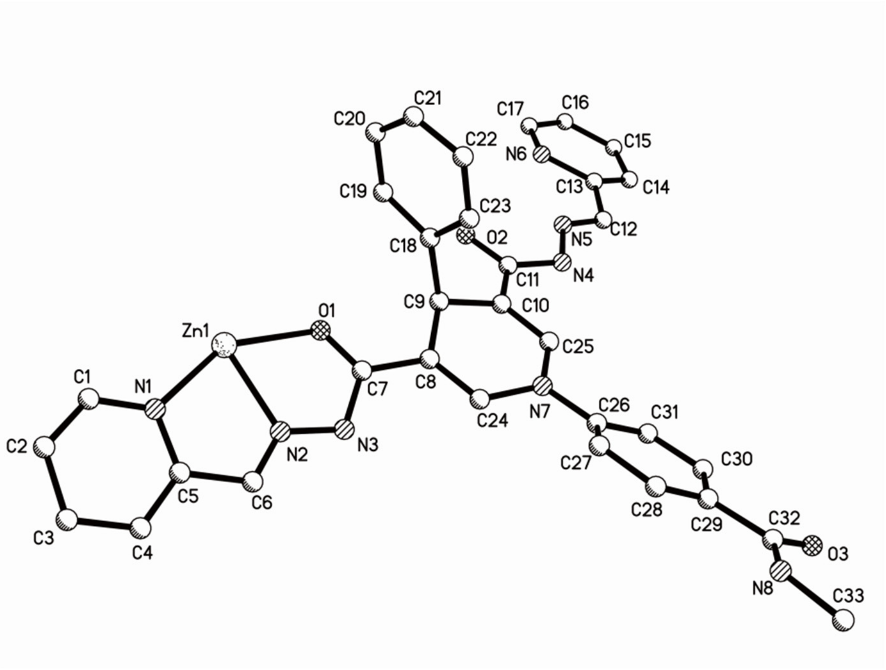

Supplementary Figure 4. Molecular structure of Zn−**ZPA** capsule within a unique asymmetric unit, showing the backbone of the ligands in the complex.

Supplementary Table 1. Selective bond distance (Å) in Zn−**ZPA**.

	bond distance (Å)		bond distance (Å)
Zn(1)-N(6)	2.049(8)	N(2)-C(6)	1.205(12)
Zn(1)-N(2)	2.076(7)	N(2)-N(3)	1.354(10)
Zn(1)-N(5)	2.091(6)	N(4)-N(5)	1.366(10)
Zn(1)-N(1)	2.101(8)	N(5)-C(12)	1.255(10)
Zn(1)-O(1)	2.172(6)	N(6)-C(17)	1.350(13)
Zn(1)-O(2)	2.197(5)	N(6)-C(13)	1.405(12)
O(1)-C(7)	1.211(9)	N(7)-C(25)	1.373(11)
O(2)-C(11)	1.235(9)	N(7)-C(24)	1.406(11)
O(3)-C(32)	1.208(12)	N(7)-C(26)	1.430(9)
N(1)-C(1)	1.289(13)	N(8)-C(32)	1.250(11)
N(1)-C(5)	1.331(12)	N(8)-C(33)	1.497(10)

Supplementary Table 2. Selective bond angle (°) in Zn−**ZPA**.

	bond angle (°)		bond angle (°)
N(6)-Zn(1)-N(2)	116.5(3)	C(11)-O(2)-Zn(1)	116.1(6)
N(6)-Zn(1)-N(5)	76.4(3)	C(1)-N(1)-C(5)	117.0(9)
N(2)-Zn(1)-N(5)	164.7(3)	C(1)-N(1)-Zn(1)	128.3(7)
N(6)-Zn(1)-N(1)	105.4(4)	C(5)-N(1)-Zn(1)	114.1(6)
N(2)-Zn(1)-N(1)	75.5(3)	C(6)-N(2)-N(3)	125.0(8)
N(5)-Zn(1)-N(1)	110.3(3)	C(6)-N(2)-Zn(1)	119.2(7)
N(6)-Zn(1)-O(1)	89.4(4)	N(3)-N(2)-Zn(1)	115.5(5)
N(2)-Zn(1)-O(1)	73.7(3)	N(2)-N(3)-C(7)	115.0(7)
N(5)-Zn(1)-O(1)	99.4(3)	N(5)-N(4)-C(11)	116.2(7)
N(1)-Zn(1)-O(1)	149.1(2)	C(12)-N(5)-N(4)	126.4(7)
N(6)-Zn(1)-O(2)	149.4(3)	C(12)-N(5)-Zn(1)	117.8(6)
N(2)-Zn(1)-O(2)	92.8(3)	N(4)-N(5)-Zn(1)	115.8(5)
N(5)-Zn(1)-O(2)	73.4(2)	C(17)-N(6)-C(13)	116.8(8)
N(1)-Zn(1)-O(2)	89.8(3)	C(17)-N(6)-Zn(1)	128.1(7)
O(1)-Zn(1)-O(2)	90.7(2)	C(13)-N(6)-Zn(1)	114.1(6)
N(6)-Zn(1)-N(2)	116.5(3)	C(7)-O(1)-Zn(1)	114.8(6)
C(7)-O(1)-Zn(1)	114.8(6)		

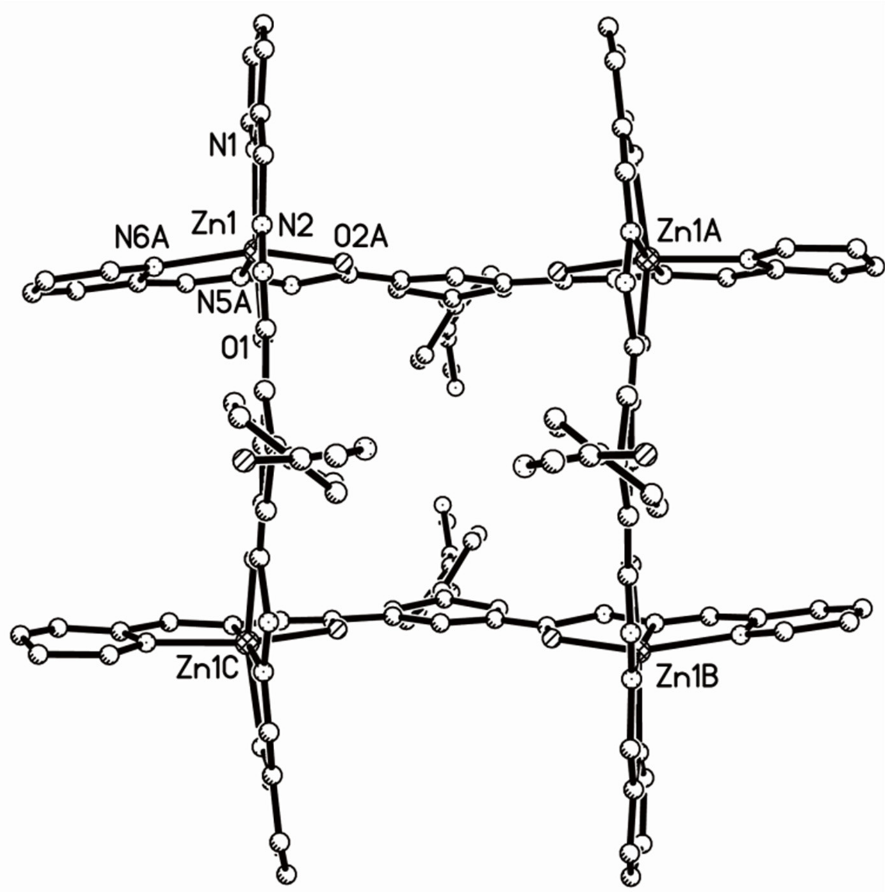

Supplementary Figure 5. A plot of the molecule Zn−**PMA** is shown. Hydrogen atoms are omitted for clarity. Symmetry code A: -y+1, x, -z+1; B: y, -x+1, -z+1; C: -x+1, -y+1, z.

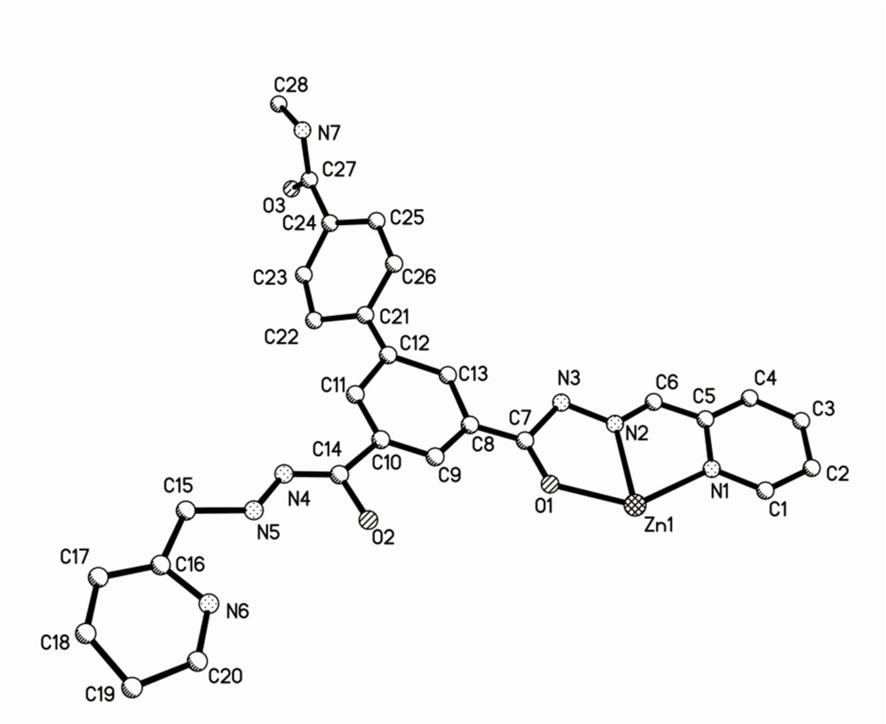

Supplementary Figure 6. Molecular structure of Zn−**PMA** capsule within a unique asymmetric unit, showing the backbone of the ligands in the complex.

Supplementary Table 3. Selective bond distance (Å) in Zn−**PMA**.

	bond distance (Å)		bond distance (Å)
Zn(1)-N(6)	2.157(13)	N(2)-C(6)	1.264(19)
Zn(1)-N(2)	2.16(2)	N(2)-N(3)	1.37(2)
Zn(1)-N(5)	2.037(18)	N(4)-N(5)	1.251(19)
Zn(1)-N(1)	2.233(11)	N(5)-C(15)	1.46(2)
Zn(1)-O(1)	2.07(1)	N(6)-C(16)	1.295(18)
Zn(1)-O(2)	2.177(13)	N(6)-C(20)	1.36(2)
O(1)-C(7)	1.244(16)	C(7)-N(3)	1.48(2)
O(2)-C(14)	1.29(2)	O(3)-C(27)	1.26(2)
N(1)-C(1)	1.341(15)	N(7)-C(27)	1.30(2)
N(1)-C(5)	1.356(17)	N(7)-C(28)	1.66(2)

Supplementary Table 4. Selective bond angle (°) in Zn−**PMA**.

	bond angle (°)		bond angle (°)
N(6)-Zn(1)-N(2)	104.2(9)	C(14)-O(2)-Zn(1)	113.1(15)
N(6)-Zn(1)-N(5)	75.6(9)	C(1)-N(1)-C(5)	118.4(11)
N(2)-Zn(1)-N(5)	174.6(6)	C(1)-N(1)-Zn(1)	121.9(12)
N(6)-Zn(1)-N(1)	98.5(5)	C(5)-N(1)-Zn(1)	118.8(11)
N(2)-Zn(1)-N(1)	69.0(7)	C(6)-N(2)-N(3)	127(2)
N(5)-Zn(1)-N(1)	105.6(6)	C(6)-N(2)-Zn(1)	117.1(17)
N(6)-Zn(1)-O(1)	96.6(4)	N(3)-N(2)-Zn(1)	115.7(12)
N(2)-Zn(1)-O(1)	74.1(6)	N(2)-N(3)-C(7)	112.3(16)
N(5)-Zn(1)-O(1)	111.3(5)	N(5)-N(4)-C(14)	126(2)
N(1)-Zn(1)-O(1)	142.5(6)	C(15)-N(5)-N(4)	130(2)
N(6)-Zn(1)-O(2)	147.7(8)	C(15)-N(5)-Zn(1)	116.0(16)
N(2)-Zn(1)-O(2)	108.1(6)	N(4)-N(5)-Zn(1)	114.3(15)
N(5)-Zn(1)-O(2)	72.2(6)	C(16)-N(6)-C(20)	119.6(18)
N(1)-Zn(1)-O(2)	91.9(5)	C(16)-N(6)-Zn(1)	122.7(18)
O(1)-Zn(1)-O(2)	93.3(4)	C(20)-N(6)-Zn(1)	117.7(17)
N(6)-Zn(1)-N(2)	104.2(9)	C(7)-O(1)-Zn(1)	121.6(13)
C(7)-O(1)-Zn(1)	121.6(13)		

ESI-MS Spectra

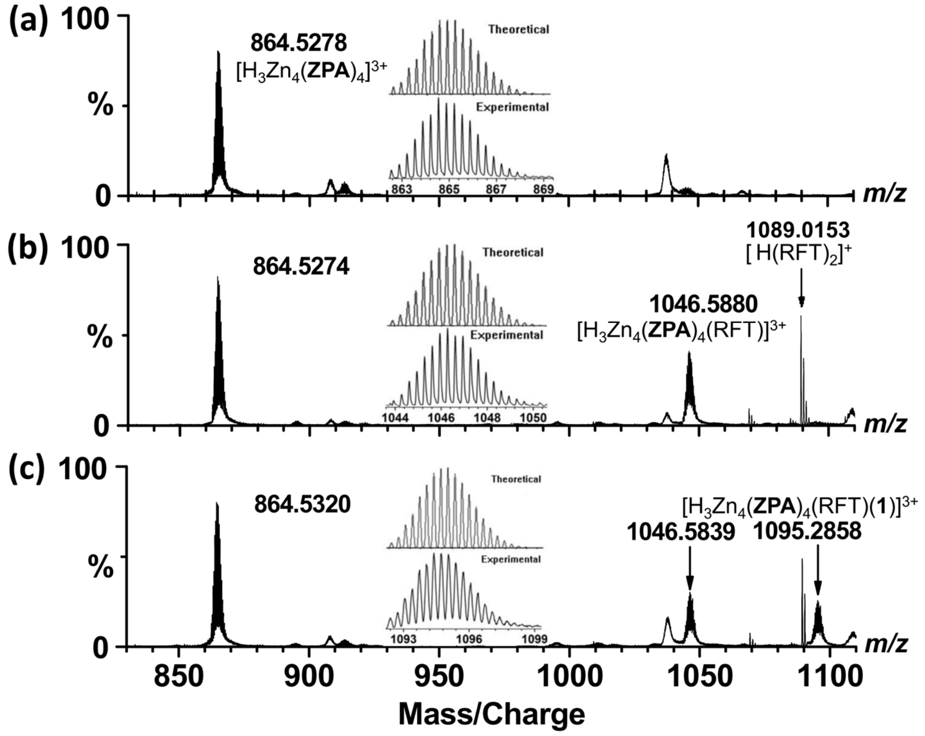

Peak	Value of m/z	Specie assigned
1	864.5278	$[H_3Zn_4(\mathbf{ZPA})_4]^{3+}$
2	1046.5880	$[H_3Zn_4(\mathbf{ZPA})_4(RFT)]^{3+}$
3	1089.0153	$[H(RFT)_2]^{+}$
4	1095.2858	$[H_3Zn_4(\mathbf{ZPA})_4(RFT)(\mathbf{1a})]^{3+}$

Supplementary Figure 7. ESI-MS spectra of Zn−**ZPA** (0.1 mM) in CH_3CN solution (a), of RFT (0.1 mM) in the aforementioned solution (b) and of **1a** (0.1 mM) and RFT (0.1 mM) in the aforementioned solution (c). The inserts show the measured and simulated isotopic patterns at *m*/*z* = 864.5278, 1046.5880, and 1095.2858, respectively.

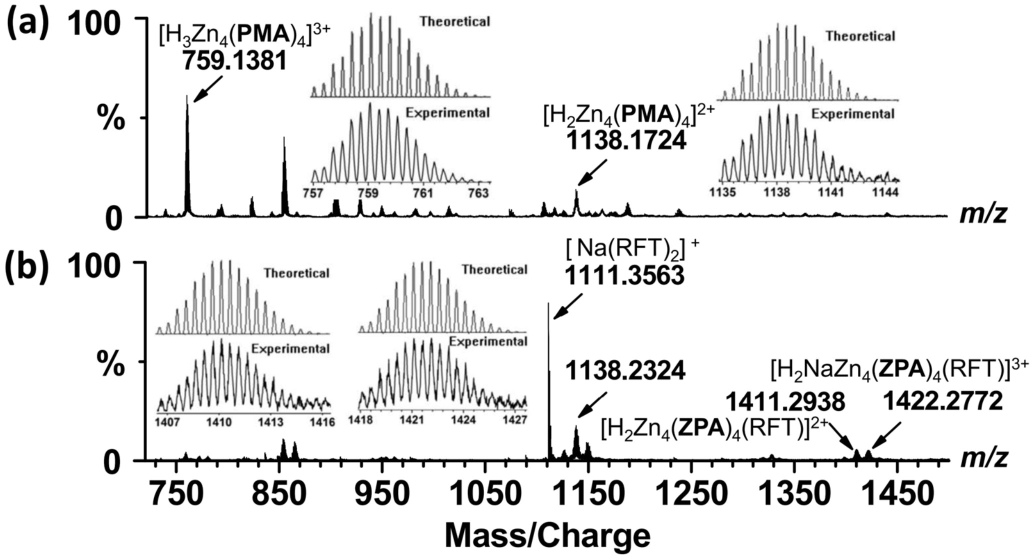

Peak	Value of m/z	Specie assigned
1	759.1381	$[H_3Zn_4(\mathbf{PMA})_4]^{3+}$
2	1111.3563	$[Na(RFT)_2]^{+}$
3	1138.1724	$[H_2Zn_4(\mathbf{PMA})_4]^{2+}$
4	1411.2938	$[H_2Zn_4(\mathbf{PMA})_4(RFT)]^{2+}$
5	1422.2772	$[HNaZn_4(\mathbf{PMA})_4(RFT)]^{2+}$

Supplementary Figure 8. ESI-MS spectra of Zn−**PMA** (0.1 mM) in CH_3CN solution (a). The measured and simulated isotopic patterns at *m*/*z* = 759.1381 and 1138.1724; ESI-MS spectra of Zn−**PMA** (0.1 mM) encapsulating RFT in CH_3CN solution (b). The measured and simulated isotopic patterns at *m*/*z* = 1112.3569, 1411.2938 and 1422.2772.

Data for Spectral Titrations

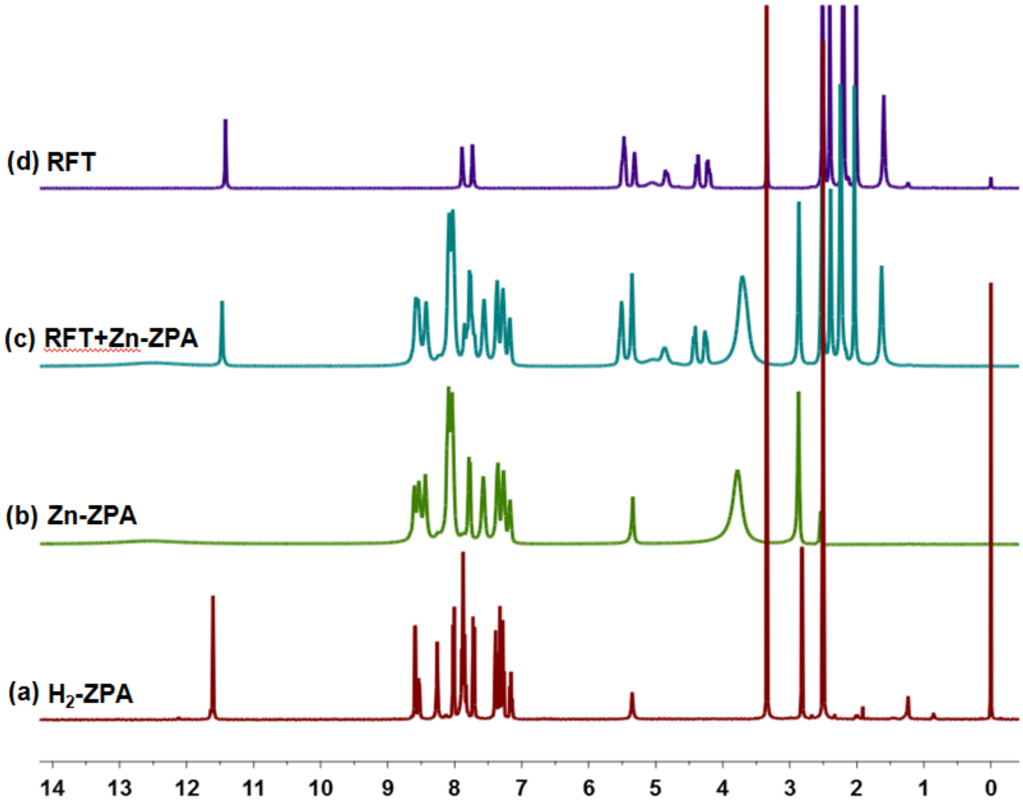

Supplementary Figure 9. ^{1}H NMR spectra of (a) the free ligand H_2**ZPA** (10.0 mM), (b) the metallacycle Zn−**ZPA** (10.0 mM), (c) Zn−**ZPA** (10.0 mM) and RFT (10.0 mM) mixture in equal concentration (d) the molecule RFT (10.0 mM) in DMSO-d_6.

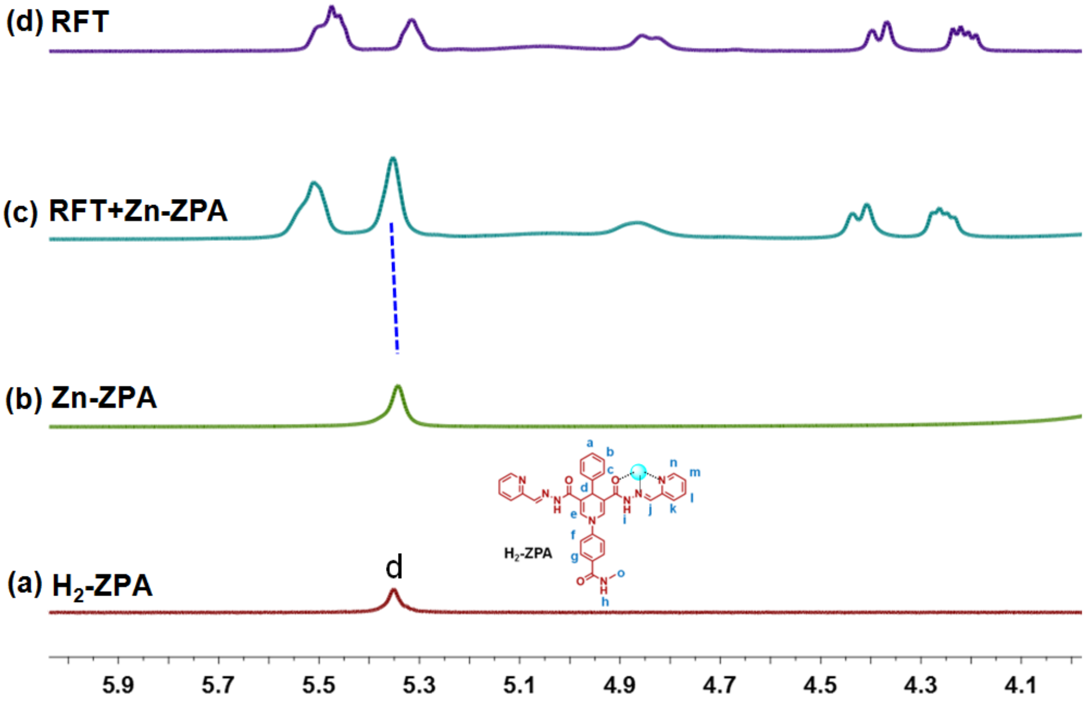

Supplementary Figure 10. Partial ^{1}H NMR spectra of (a) the free ligand H2**ZPA** (10.0 mM), (b) the metallacycle Zn−**ZPA** (10.0 mM), (c) Zn−**ZPA** (10.0 mM) and RFT (10.0 mM) mixture in equal concentration (d) the molecule RFT (10.0 mM) in DMSO-d_6.

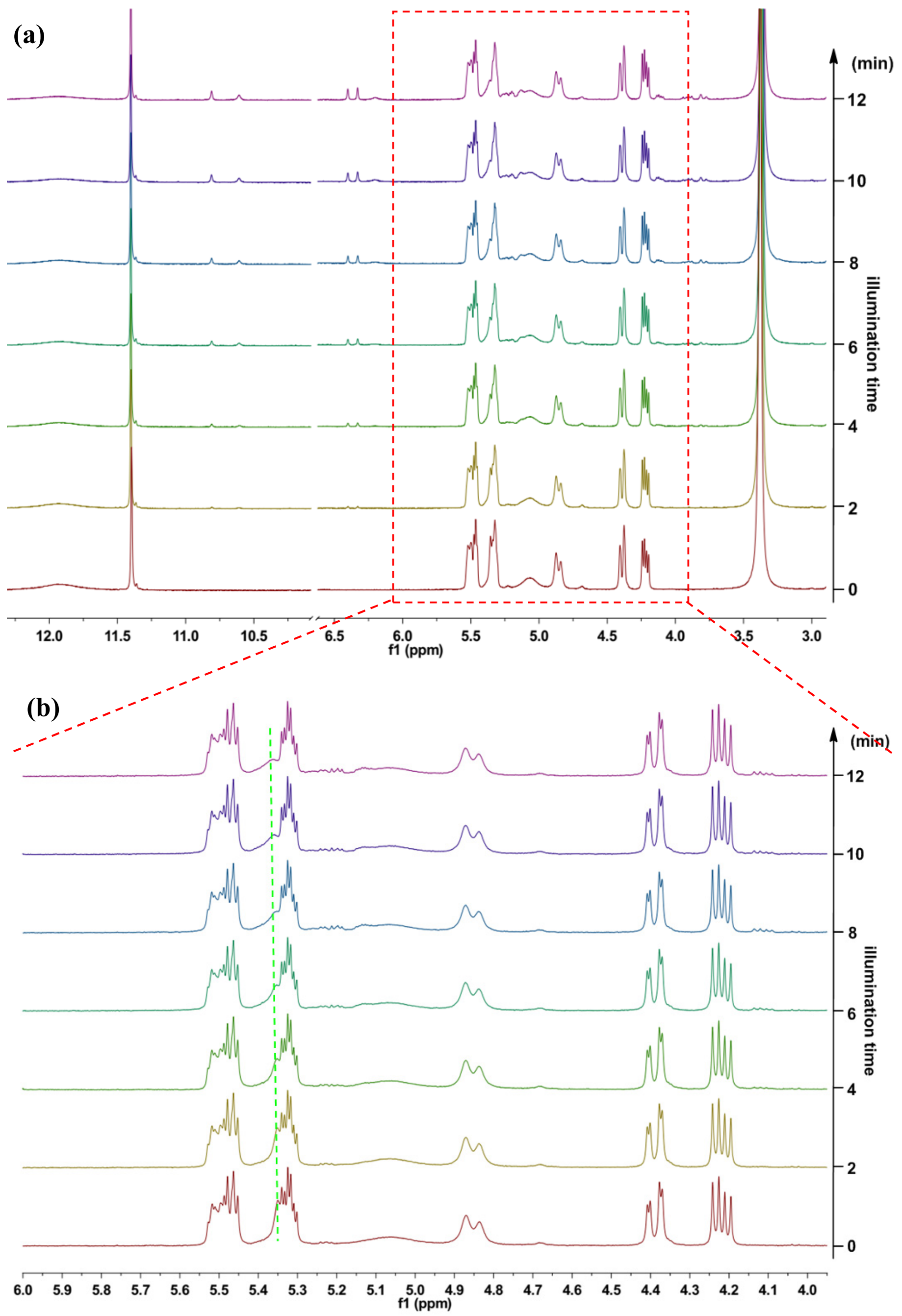

Supplementary Figure 11. (a) Partial ^{1}H NMR spectra recorded before and during the reaction of Zn−**ZPA** (10.0 mM) and RFT (10.0 mM) with illumination (455 nm) in deaerated DMSO-d_6 on the timescale. (b) Enlarged view of the ^{1}H NMR spectra mentioned above, showing that the peaks of the NADH active sites on Zn−**ZPA** at approximately 5.35 ppm gradually disappeared as the reaction progressed.

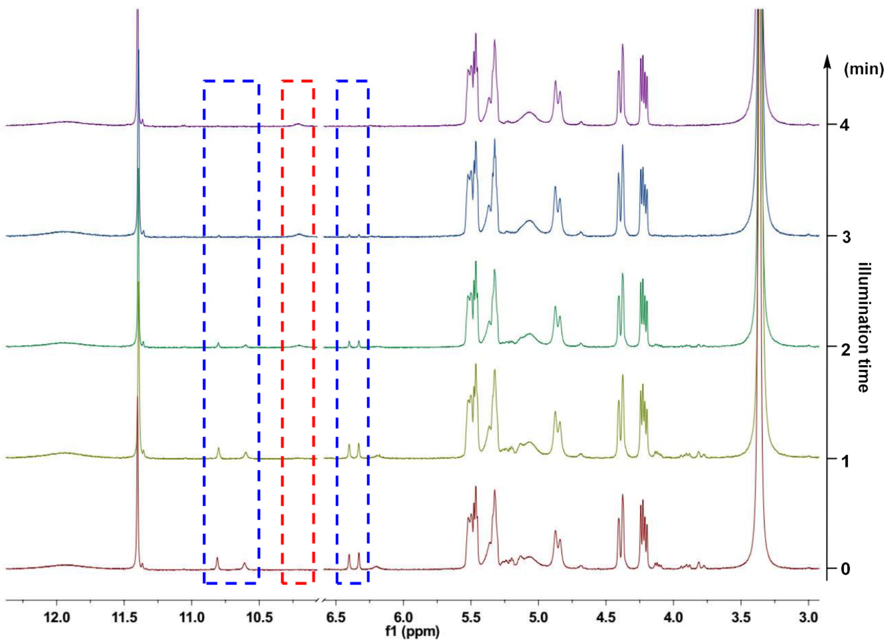

Supplementary Figure 12. Partial ^{1}H NMR spectra recorded when the system mentioned above (Supplementary Figure 11) exposed to oxygen in DMSO-d_6 on the timescale.

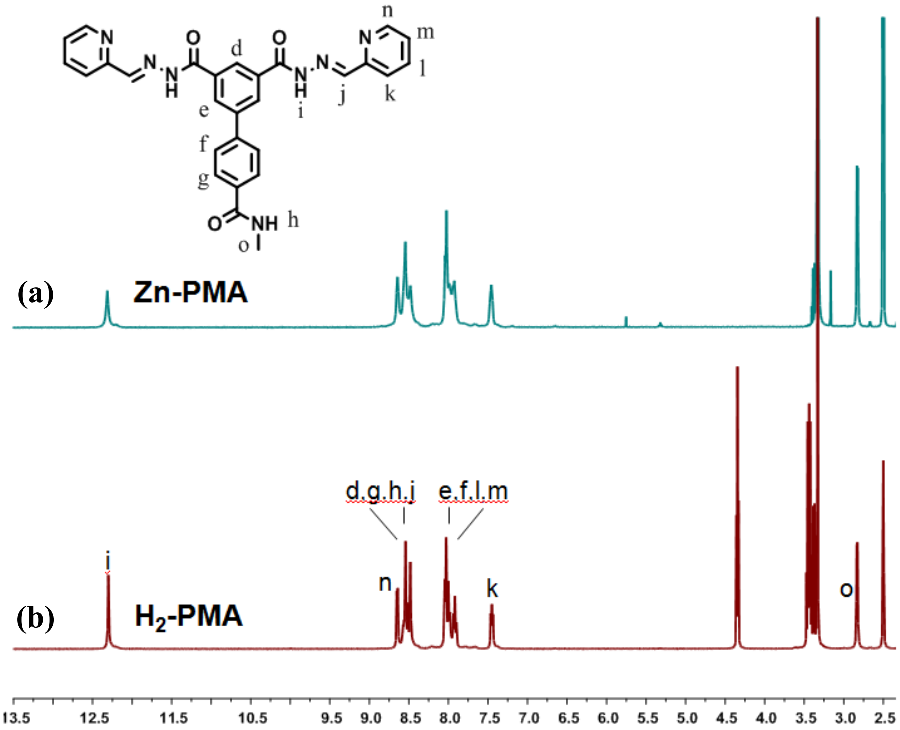

Supplementary Figure 13. ^{1}H NMR spectra of (a) the metallacycle Zn−**PMA** (1.0 mM), (b) H_2**PMA** (1.0 mM) in DMSO-d_6.

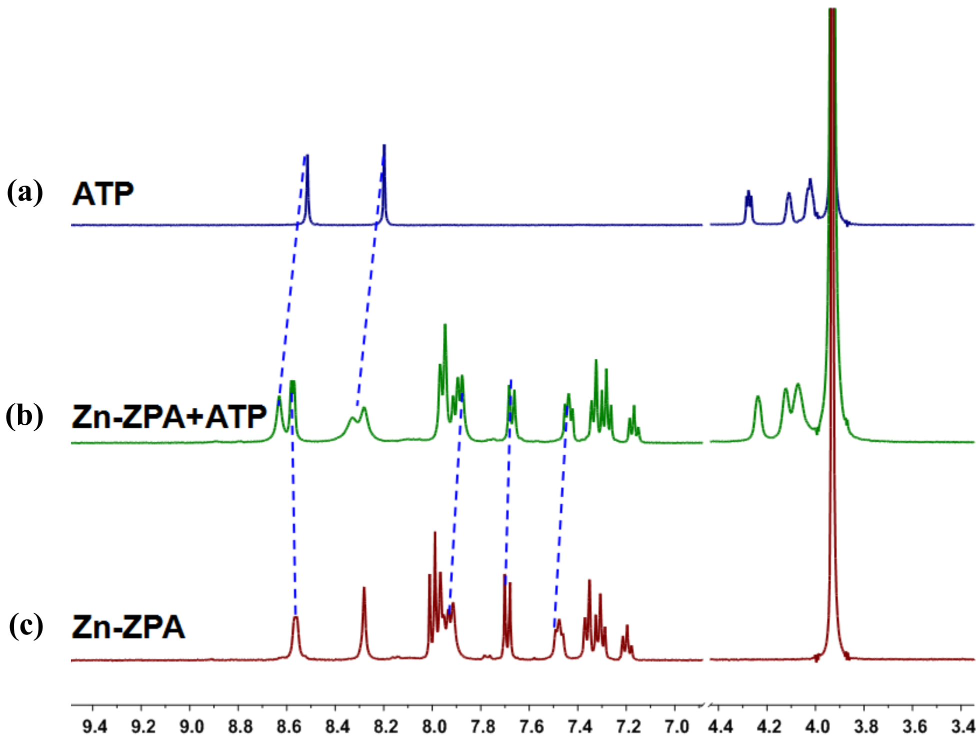

Supplementary Figure 14. Partial ^{1}H NMR spectra of (a) **ATP** (1.0 mM), (b) the metallacycle Zn−**ZPA** (1.0 mM), (c) Zn−**ZPA** (1.0 mM) and **ATP** in equal concentration in DMSO-d_6/D_2O (v/v=5/1).

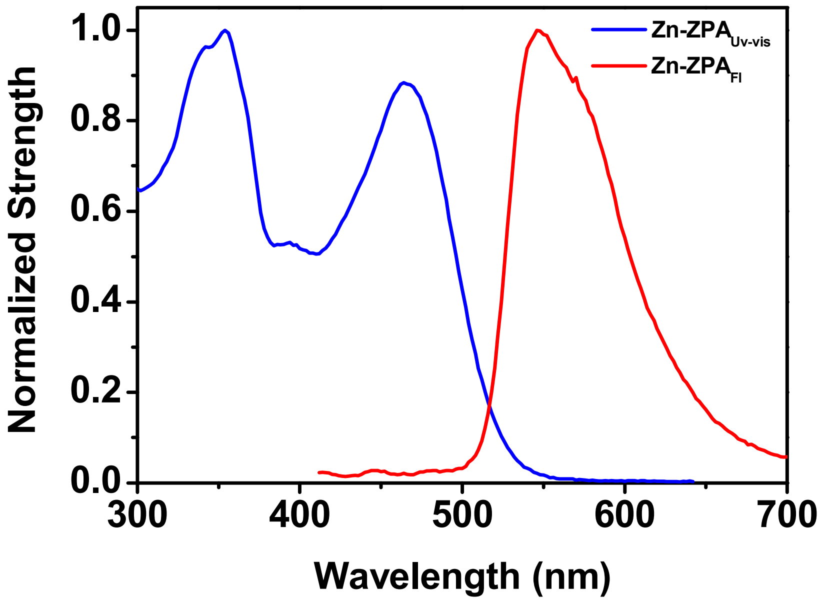

Supplementary Figure 15. Normalized UV-Vis absorption and emission spectrum of Zn−**ZPA** (10.0 μM) in CH_3CN.

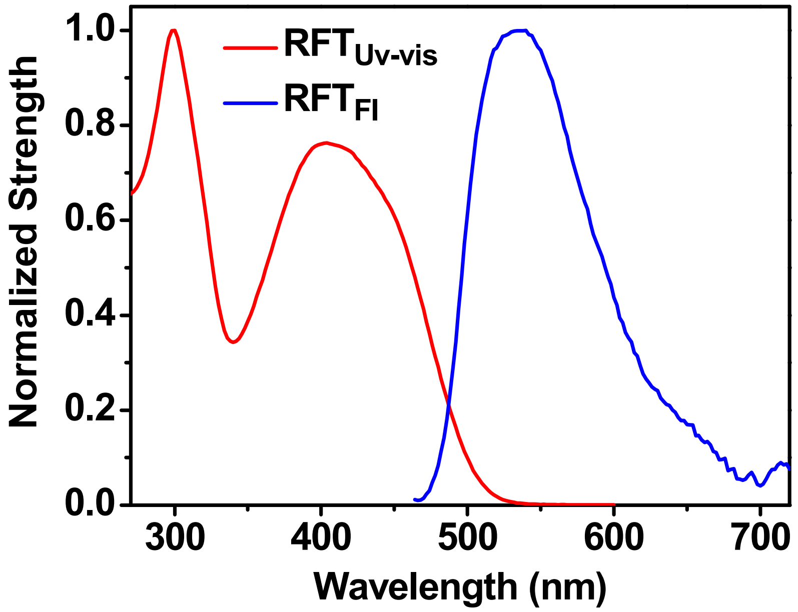

Supplementary Figure 16. Normalized UV-Vis absorption and emission spectrum of RFT (10.0 μM) in CH_3CN.

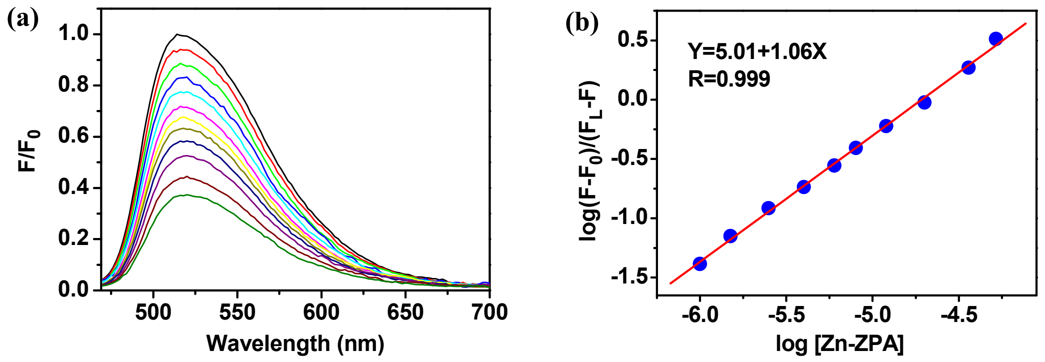

Supplementary Figure 17. (a) Family of luminescence spectra of RFT (10.0 μM) in CH_3CN/H_2O (v/v = 2/1) upon addition of Zn−**ZPA** (50.0 μM) with various concentration. (b) The Hill-plot of titration curve. Fluorescence intensity was recorded at 535 nm, excited at 455 nm.

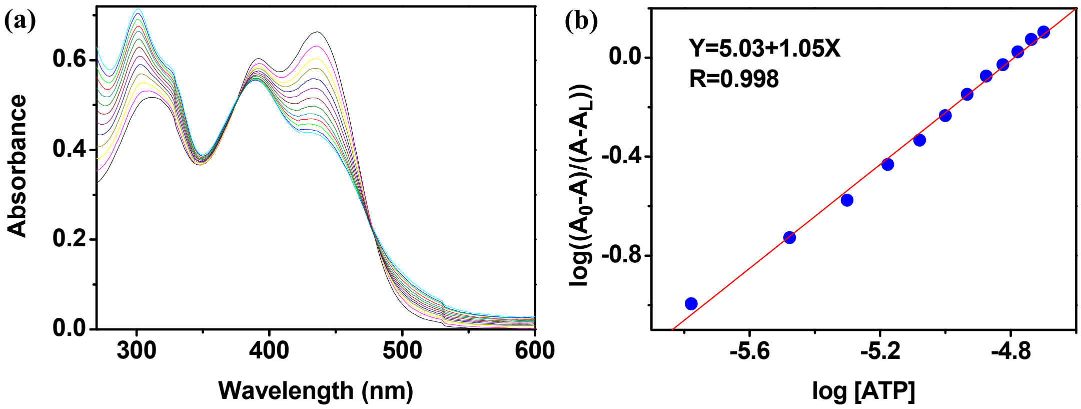

Supplementary Figure 18. (a) Family of UV-Vis absorption spectra of Zn−**ZPA** (10.0 μM) in CH_3CN/H_2O (v/v = 2/1) upon addition of ATP (total 20.0 μM) with various concentration. (b) Linear fitting of the corresponding curves showing the calculation of the associate constant.

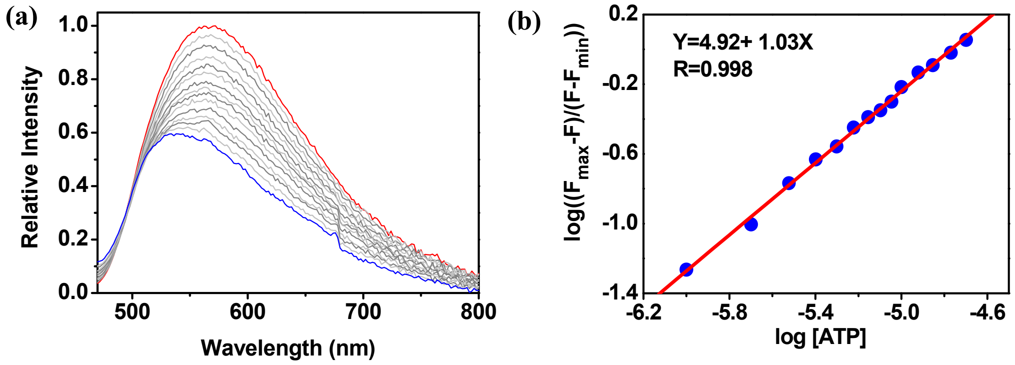

Supplementary Figure 19. (a) Family of luminescence spectra of Zn−**ZPA** (10.0 μM) in CH_3CN/H_2O (v/v = 2/1) upon the addition of ATP (total 10.0 μM). (b) The Hill-plot of titration curve. Fluorescence intensity was recorded at 565 nm.

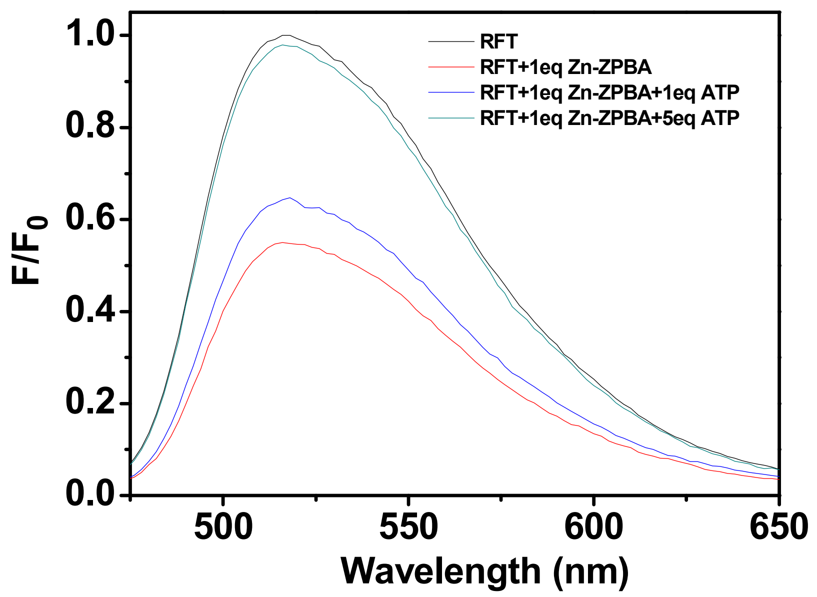

Supplementary Figure 20. Family of luminescence spectra of RFT (10.0 μM) in CH_3CN/H_2O (v/v = 2/1) upon the addition of Zn−**ZPA** (10.0 μM), of both Zn−**ZPA** (10.0 μM) and ATP (10.0 μM or 50.0 μM). Fluorescence intensity was excited at 455 nm.

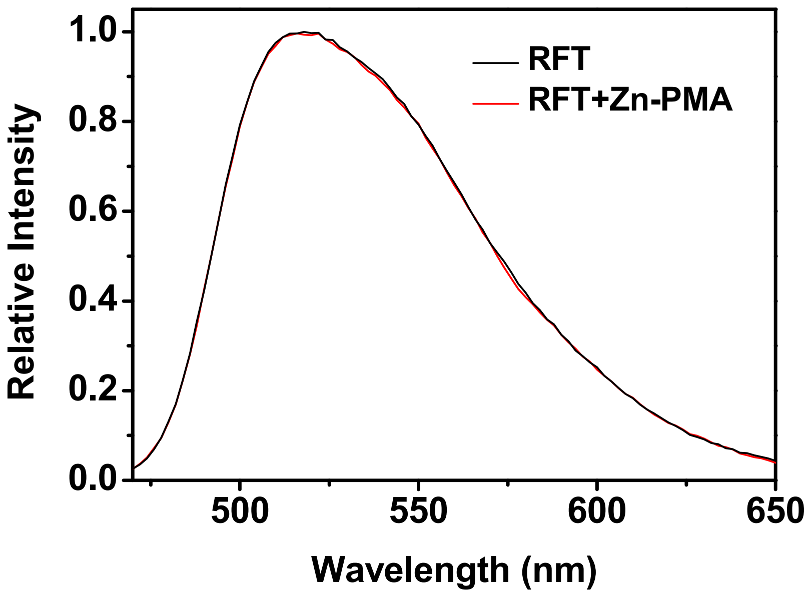

Supplementary Figure 21. Family of luminescence spectra of RFT (10.0 μM) in CH_3CN/H_2O (v/v = 2/1) upon the addition of Zn−**PMA** (10.0 μM). Fluorescence intensity was excited at 455 nm.

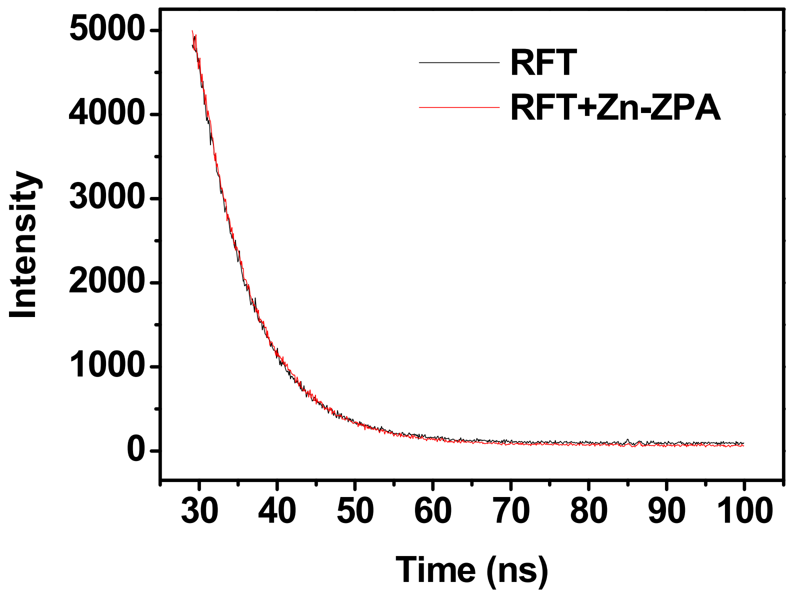

Supplementary Figure 22. Luminescence decay of RFT (10.0 μM, red line) in CH_3CN and of the aforementioned solution upon addition of Zn−**ZPA** (10.0 μM). The intensity was recorded at 540 nm and the excitation wavelength was 472 nm.

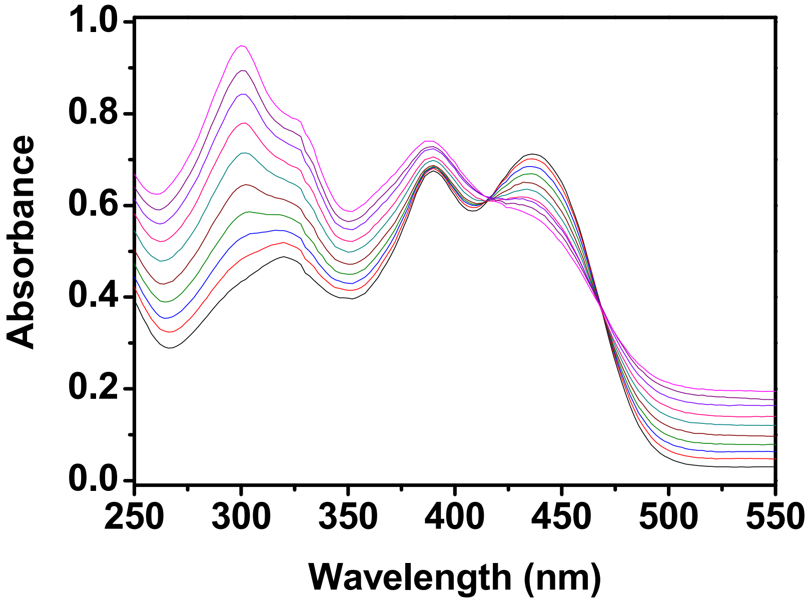

Supplementary Figure 23. Family of UV-Vis absorption spectra of Zn−**ZPA** (10.0 μM) in CH_3CN/H_2O (v/v = 2/1) upon addition of FDH (total 1.0 μM) with various concentration.

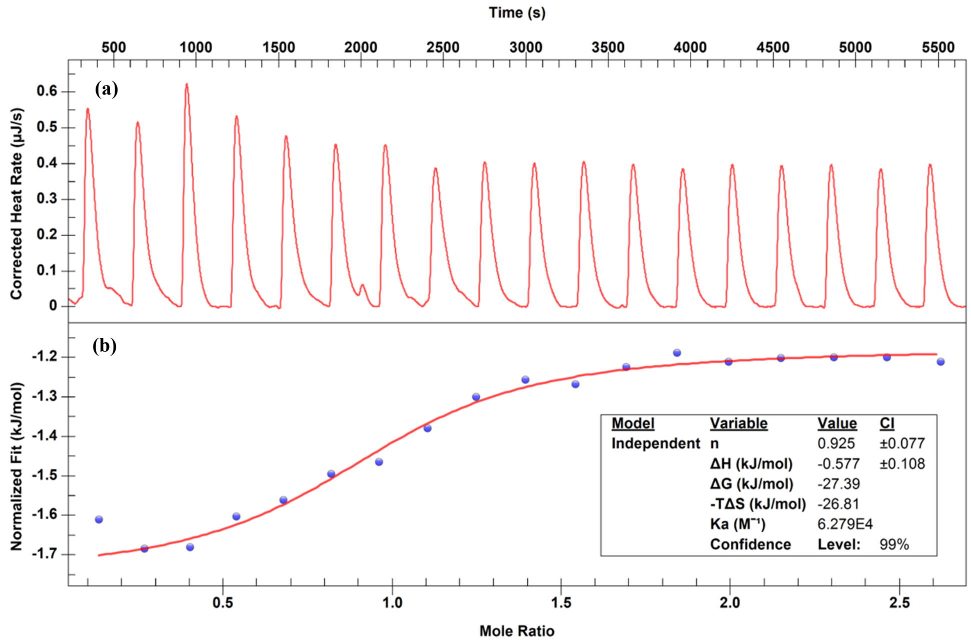

Supplementary Figure 24. The ITC experiments were performed by an isothermal titration microcalorimeter at atmospheric pressure and at 25.0ºC, giving the association constants (K) and the thermodynamic parameters. A solution of guest in a 0.25 mL syringe was sequentially injected with stirring at 250 rpm into a solution of host in the sample cell (1.30 mL volume). All the thermodynamic parameters reported in this work were obtained by using the 'independent' model.

Microcalorimetric titration of Zn−**ZPA** with RFT in CH_3CN/H_2O (v/v, 2/1) solution at 298.15K. (a) Raw data for sequential 25 injections (10.0 µL per injection) of RFT solution (2.5 mM) injecting into Zn−**ZPA** solution (0.2 mM). (b) Apparent reaction heat obtained from the integration of calorimetric traces.

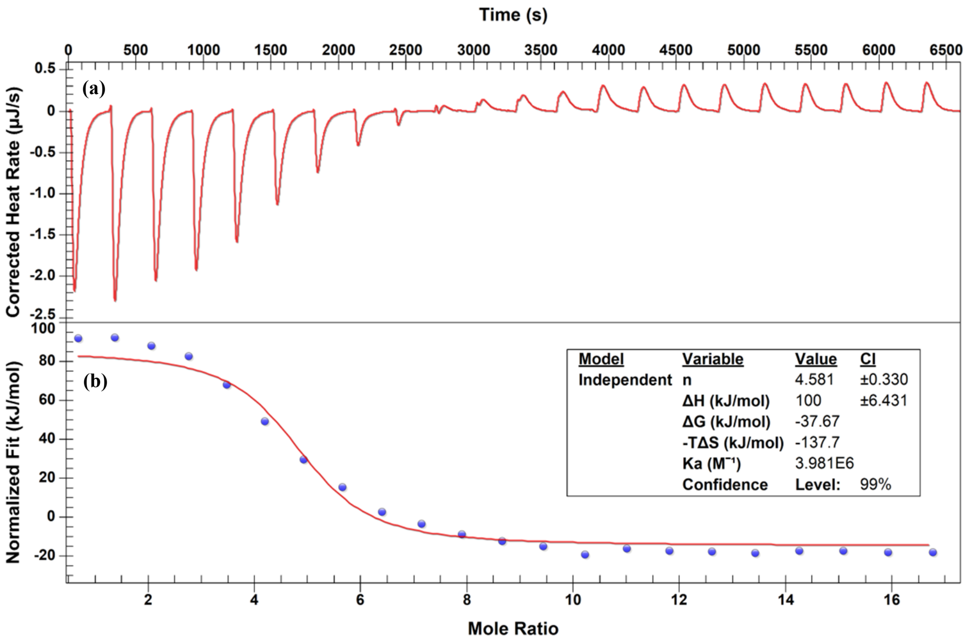

Supplementary Figure 25. The ITC experiments were performed by an isothermal titration microcalorimeter at atmospheric pressure and at 25.0ºC, giving the association constants (K) and the thermodynamic parameters. A solution of guest in a 0.25 mL syringe was sequentially injected with stirring at 250 rpm into a solution of host in the sample cell (1.30 mL volume). All the thermodynamic parameters reported in this work were obtained by using the 'independent' model.

Microcalorimetric titration of enzyme FDH with Zn−**ZPA** in CH_3CN/H_2O (v/v, 2/1) solution at 298.15K. (a) Raw data for sequential 25 injections (10.0 µL per injection) of Zn−**ZPA** solution (0.16 mM) injecting into FDH solution (2.5 µM). (b) Apparent reaction heat obtained from the integration of calorimetric traces.

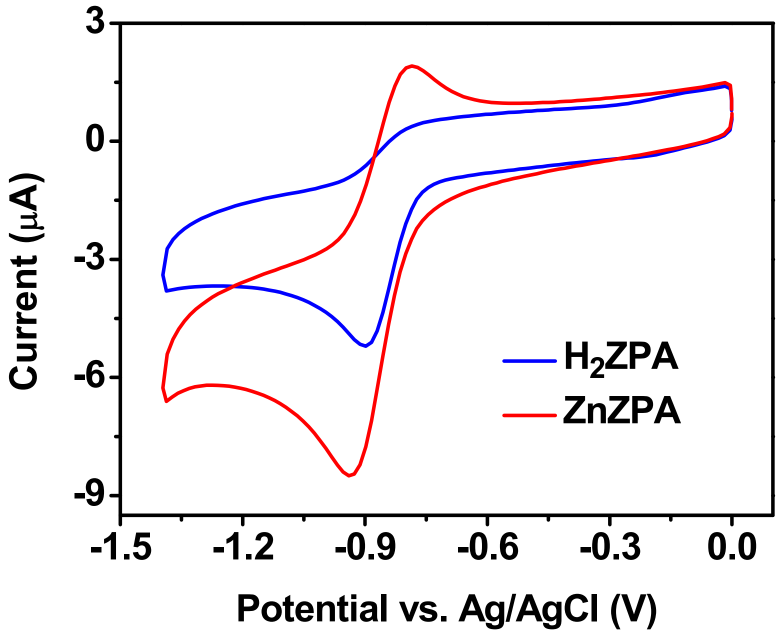

Supplementary Figure 26. Cyclic voltammogram of Zn−**ZPA** (0.1 mM) and H_2**ZPA** (0.4 mM) in CH_3CN containing $TBAPF_6$ (0.1 M). Scan Rate: 100 mV/s.

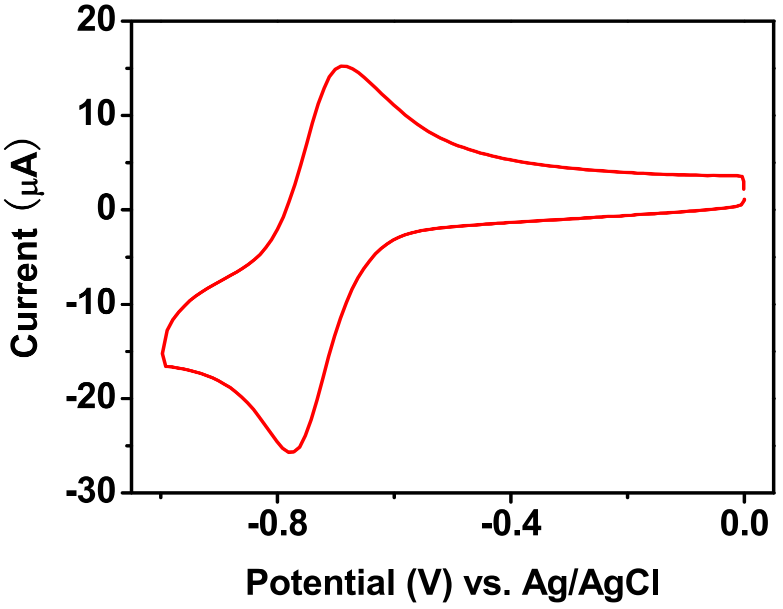

Supplementary Figure 27. Cyclic voltammograms of RFT (0.1 mM) in CH_3CN containing $TBAPF_6$ (0.1 M). Scan Rate: 100 mV/s.

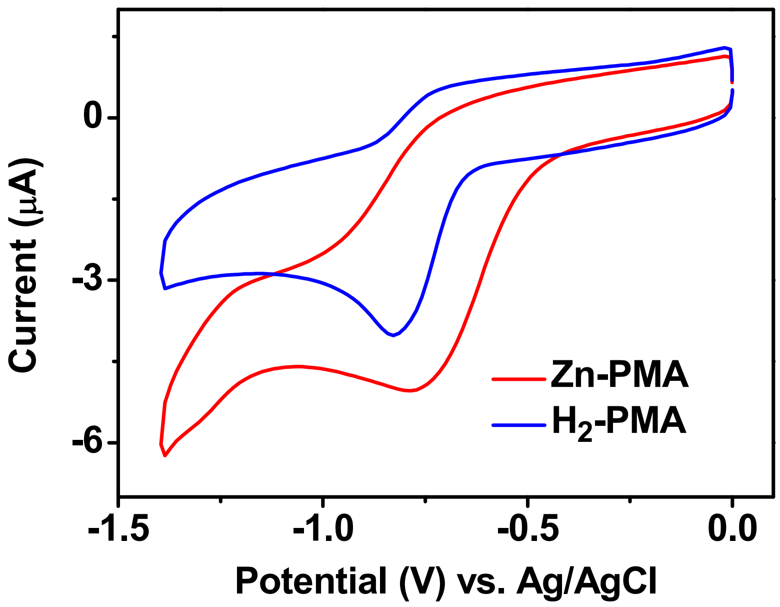

Supplementary Figure 28. Cyclic voltammogram of Zn−**PMA** (0.1 mM) and H_2**PMA** (0.4 mM) in CH_3CN containing $TBAPF_6$ (0.1 M). Scan Rate: 100 mV/s.

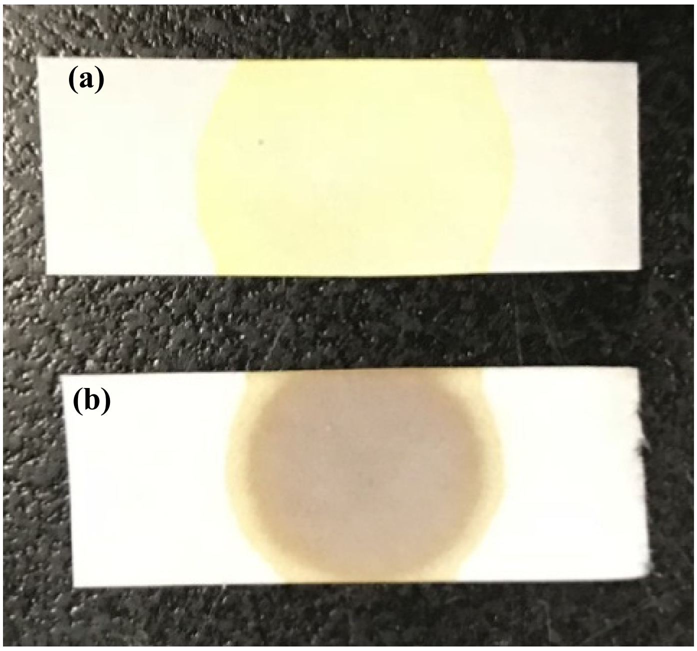

Supplementary Figure 29. The potassium iodide starch test paper was used to visually detect the peroxide in the reaction: (a) before reaction; (b) during reaction.

Data Relative to Monooxygenation

Supplementary Table 5. The Baeyer-Villiger Oxidation of 3-phenylcyclobutanone (**1a**) in the presence of oxygen.

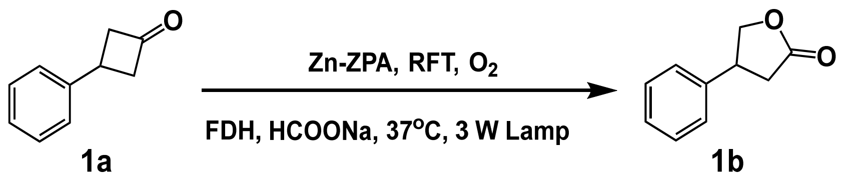

Entry	Cofactor	Enzyme	Inhibitor	Yield (%)
1	Zn−**ZPA**	FDH	None	87
2	Zn−**ZPA**	None	None	15
3	Zn−**ZPA**	FDH	ATP	18

Reaction conditions: CH_3CN/PBS (2:1), **1a** (8.0 mM), Zn−**ZPA (**1.6 mM**)**, RFT (1.6 mM), FDH (2 U·mL^{-1}), HCOONa (16.0 mM), ATP (16.0 mM), O_2, 3 W fluorescent lamp, 37ºC, 18 h. Yields were determined by integral area of ^{1}H NMR with internal standards.

General methods for catalytic Baeyer-Villiger reactions

Zn−**ZPA** with a magnetic stir bar in 5 mL CH_3CN/PBS (2/1, pH 7.40) added in a 15 mL flask. Then, RFT combined with HCOONa (16.0 mM)/ CH_3CH_2OH (3% in volumn) added to the solution containing Zn−**ZPA**. The flask was full with O_2 by bubbling oxygen for 20 min under atmospheric pressure. A second stock solution of FDH /ADH in buffer solution was added to the above reaction vial. Subsequently, substrate was added by syringe. The oxygen in reaction vial was adequate with an oxygen balloon. The reaction vial was used a constant temperature water circulation device to maintain the temperature at 37ºC. And the vial was irradiated with fluorescent lamp (3 W) with constant stirring for 9 h. And then, additional FDH /ADH was added to the final volume of the reaction mixture and the resulting solution was kept stirring gently for another 9 h. The ^{1}H NMR spectrums were recorded to determine the yields (relative to the remaining starting material or the internal standard mesitylene benzene). The crude product could be purified by column chromatography on silica gel to afford the corresponding products.

General methods for catalytic monooxygenation of thioethers

Zn−**ZPA** with a magnetic stir bar in 5 mL CH_3CN/PBS (2/1, pH 7.40) added in a 15 mL flask. Then, RFT combined with HCOONa (16.0 mM)/ CH_3CH_2OH (3% in volumn) added to the solution containing Zn−**ZPA**. The flask was full with O_2 by bubbling oxygen for 20 min under atmospheric pressure. A second stock solution of FDH/ADH in buffer solution was added to the above reaction vial. Subsequently, substrate was added by syringe. The oxygen in reaction vial was adequate with an oxygen balloon. The reaction vial was used a constant temperature water circulation device to maintain the temperature at 37ºC. And the vial was irradiated with fluorescent lamp (3 W) with constant stirring for 4 h. The ^{1}H NMR spectrums were recorded to determine the yields (relative to the remaining starting material or the internal standard mesitylene benzene). The crude product could be purified by column chromatography on silica gel to afford the corresponding products.

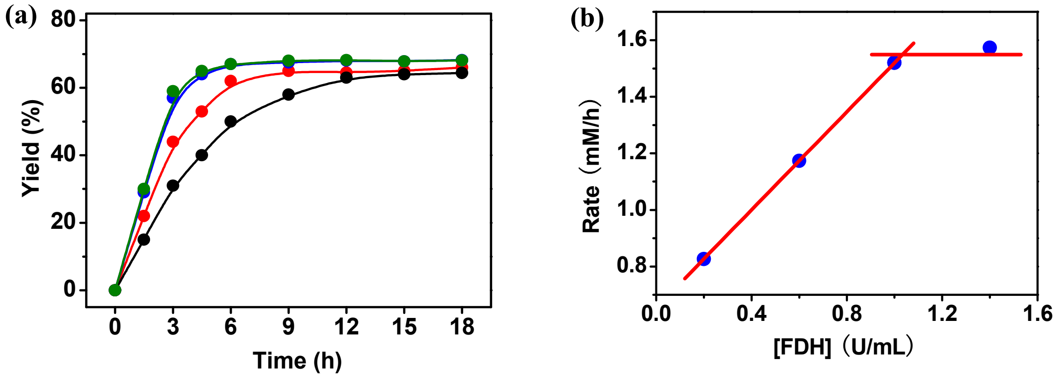

Supplementary Figure 30. (a) Kinetics of the Baeyer-Villiger oxidation. **1a** (8.0 mM), Zn−**ZPA** (1.6 mM), RFT (1.6 mM) and HCOONa (16.0 mM) with different concentration of FDH in a CH_3CN/PBS solution (2:1 v/v), 3 W fluorescent lamp, 18 h. (b) The picture showing the initial rate *vs*. concentration of the FDH.

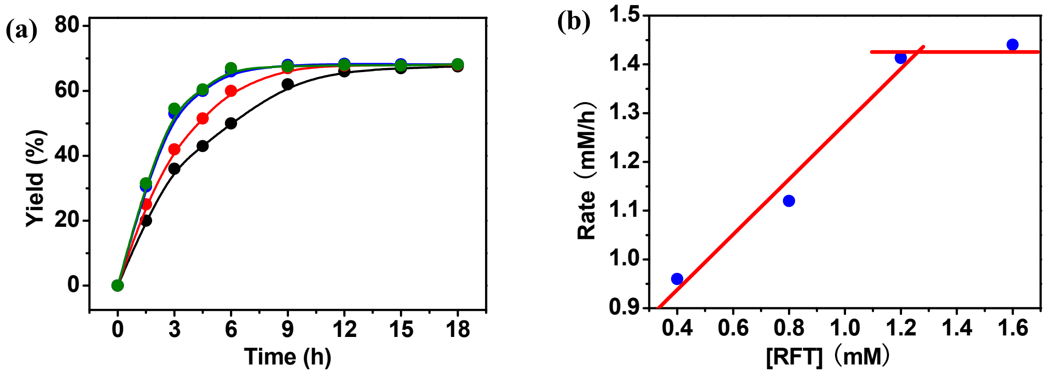

Supplementary Figure 31. (a) Kinetics of the Baeyer-Villiger oxidation. **1a** (8.0 mM), Zn−**ZPA** (1.6 mM), FDH (1 U·mL^{-1}) and HCOONa (16.0 mM) with different concentration of RFT in a CH_3CN/PBS solution (2:1 v/v), 3 W fluorescent lamp, 18 h. (b) The picture showing the initial rate *vs*. concentration of the RFT.

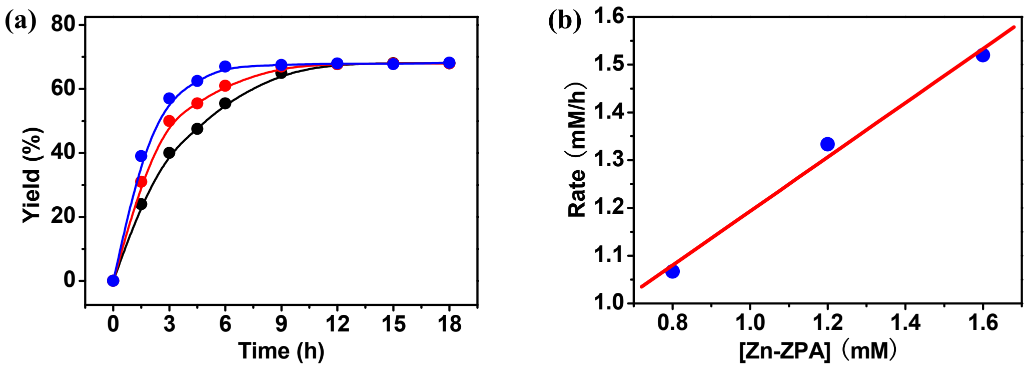

Supplementary Figure 32. (a) Kinetics of the Baeyer-Villiger oxidation. **1a** (8.0 mM), RFT (1.6 mM), FDH (1 U·mL^{-1}) and HCOONa (16.0 mM) with different concentration of Zn−**ZPA** in a CH_3CN/PBS solution (2:1 v/v), 3 W fluorescent lamp, 18 h. (b) The picture showing the initial rate *vs*. concentration of the Zn−**ZPA**.

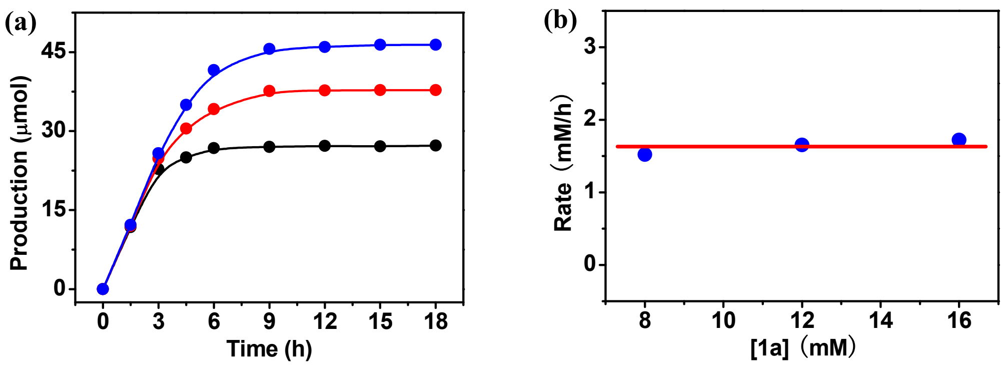

Supplementary Figure 33. (a) Kinetics of the Baeyer-Villiger oxidation. Zn−**ZPA** (1.6 mM), RFT (1.6 mM), FDH (1 U·mL^{-1}) and HCOONa (16.0 mM) with different concentration of **1a** in a CH_3CN/PBS solution (2:1 v/v), 3 W fluorescent lamp, 18 h. (b) The picture showing the initial rate *vs*. concentration of **1a**.

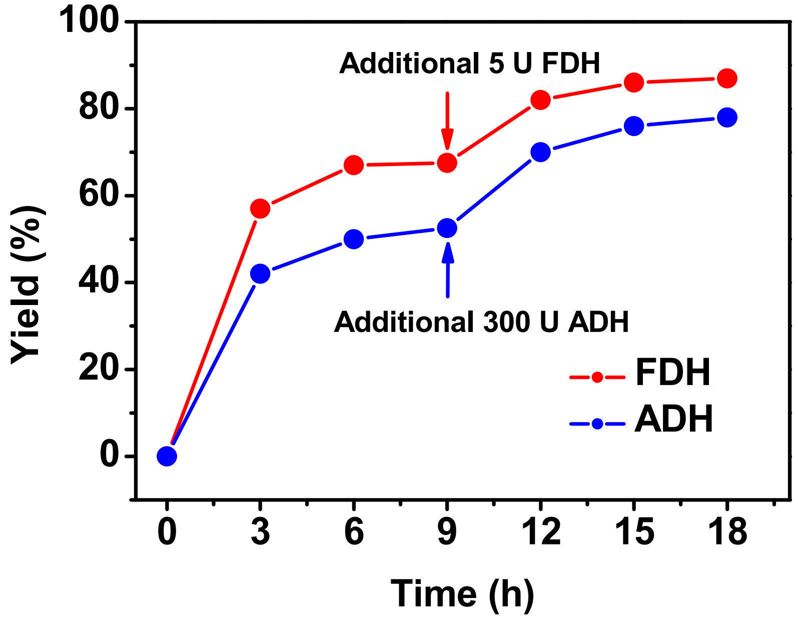

Supplementary Figure 34. Kinetics of the Baeyer-Villiger oxidation. **1a** (8.0 mM), Zn−**ZPA** (1.6 mM), RFT (1.6 mM), FDH (2 U·mL^{-1}) and HCOONa (16.0 mM) in a CH_3CN/PBS solution (2:1 v/v), 3 W fluorescent lamp, 18 h (red). The yields by ADH (120 U·mL^{-1}) and CH_3CH_2OH (3% in volume) as alternatives for FDH and HCOONa were shown in blue. FDH or ADH was added again (total 2 U·mL^{-1}) after 9 hours.

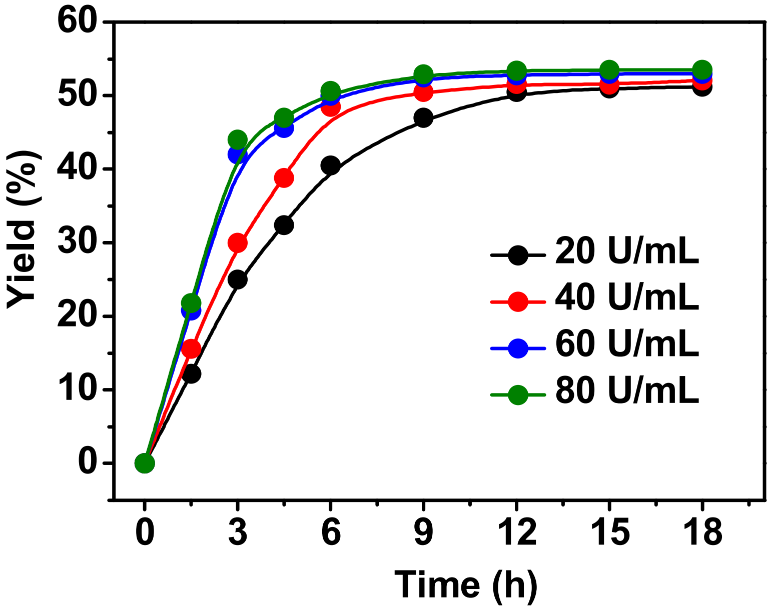

Supplementary Figure 35. Kinetics of the Baeyer-Villiger oxidation of **1a** in Zn−**ZPA**/RFT/ADH system, showing yield of **1b** as the concentration of ADH varies with Zn−**ZPA** (1.6 mM), RFT (1.6 mM) and **1a** (8.0 mM) fixed in the system containing 3% CH_3CH_2OH (in volume) in a CH_3CN/PBS solution (2:1) with 3 W fluorescent lamp in the presence of oxygen.

General methods for theoretical 'docking study'

Docking calculations were performed with the AutoDock program 4.2. The metal-organic capsule Zn−**ZPA** and RFT were downloaded from the CCDC database, the enzyme Formate Dehydrogenase were downloaded from the PDB database. The models of the enzyme and organic molecules were refined by removing hydrogen atoms. Polar hydrogens were then added, followed by assignment of Kollman charges, fragmental volumes, and atomic solvation parameters to adhesive by means of AutoDock Tools. For the ligand, the molecule was refined by removing and subsequently adding hydrogen atoms in a similar manner to that for adhesive. Next, Gasteiger partial charges were assigned to the ligands, and nonpolar hydrogens were merged. All torsions were allowed to rotate during docking. The Lamarckian genetic algorithm was used to determine the appropriate binding positions, orientations, and conformations of the ligands. Default parameters were used, except for the number of generations which was set to 300. The best docking mode of the receptor-ligand complex was chosen based on the binding energy score, clustering, and chemical reasonableness.

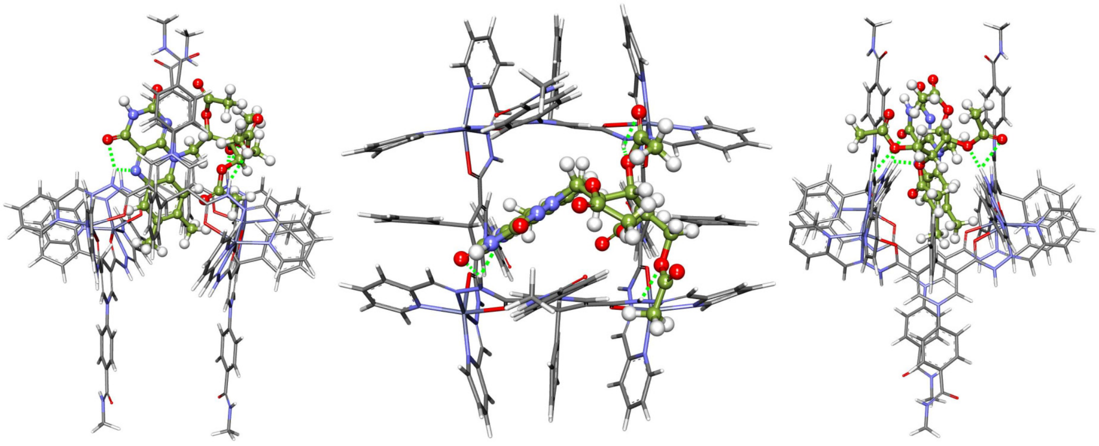

Supplementary Figure 36. Theoretical 'docking study' optimized model of Zn−**ZPA** ⊃ RFT.

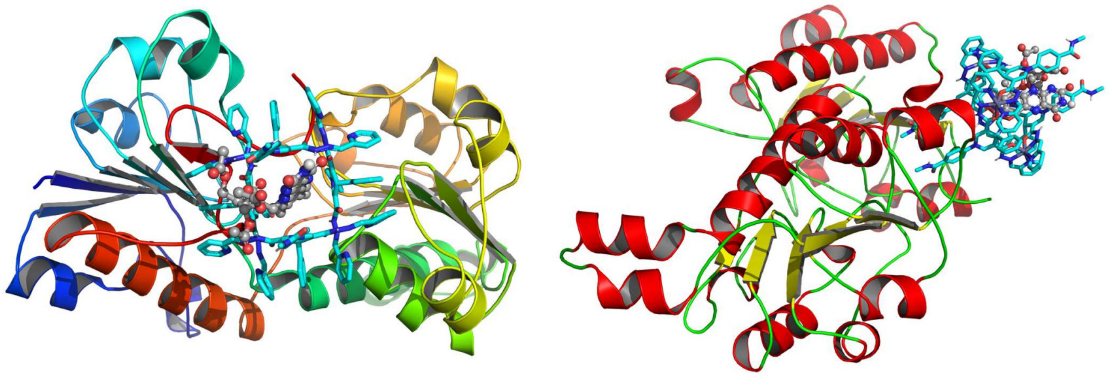

Supplementary Figure 37. Theoretical 'docking study' optimized model of Zn−**ZPA** ⊃ RFT with enzyme FAD.

^{1}H NMR Date for substrates and product from the biomimetic catalysis

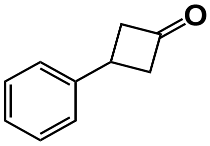

3-Phenylcyclobutanone (1a): styrene (1.21 g, 11.6 mmol) was used as raw material, yielding colorless oil. Yield: 1.02 g (60%). ^{1}H NMR (400 MHz, $CDCl_3$, ppm): *δ* 7.37-7.24 (m, 5H), 3.71-3.65 (m, 1H), 3.53-3.46 (m, 2H), 3.29-3.22 (m, 2H).

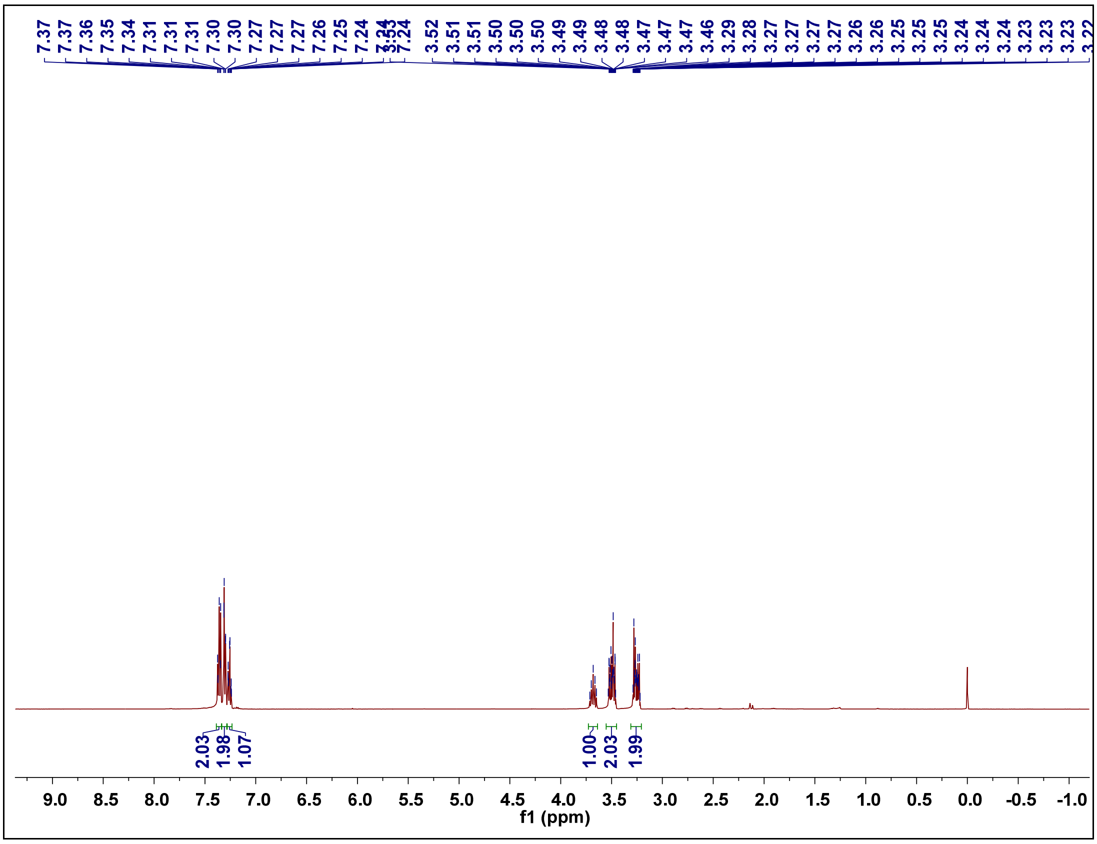

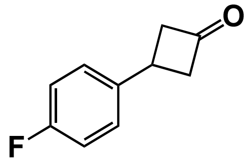

3-(4-fluorophenyl)cyclobutanone (2a): 1-fluoro-4-vinylbenzene (1.42 g, 11.6 mmol) was used as raw material, yielding colorless oil. Yield: 0.67 g (42%). ^{1}H NMR (400 MHz, $CDCl_3$, ppm): δ 7.30-7.26 (m, 2H), 7.08-7.04 (m, 2H), 3.73-3.65 (m, 1H), 3.56-3.48 (m, 2H), 3.28-3.19 (m, 2H).

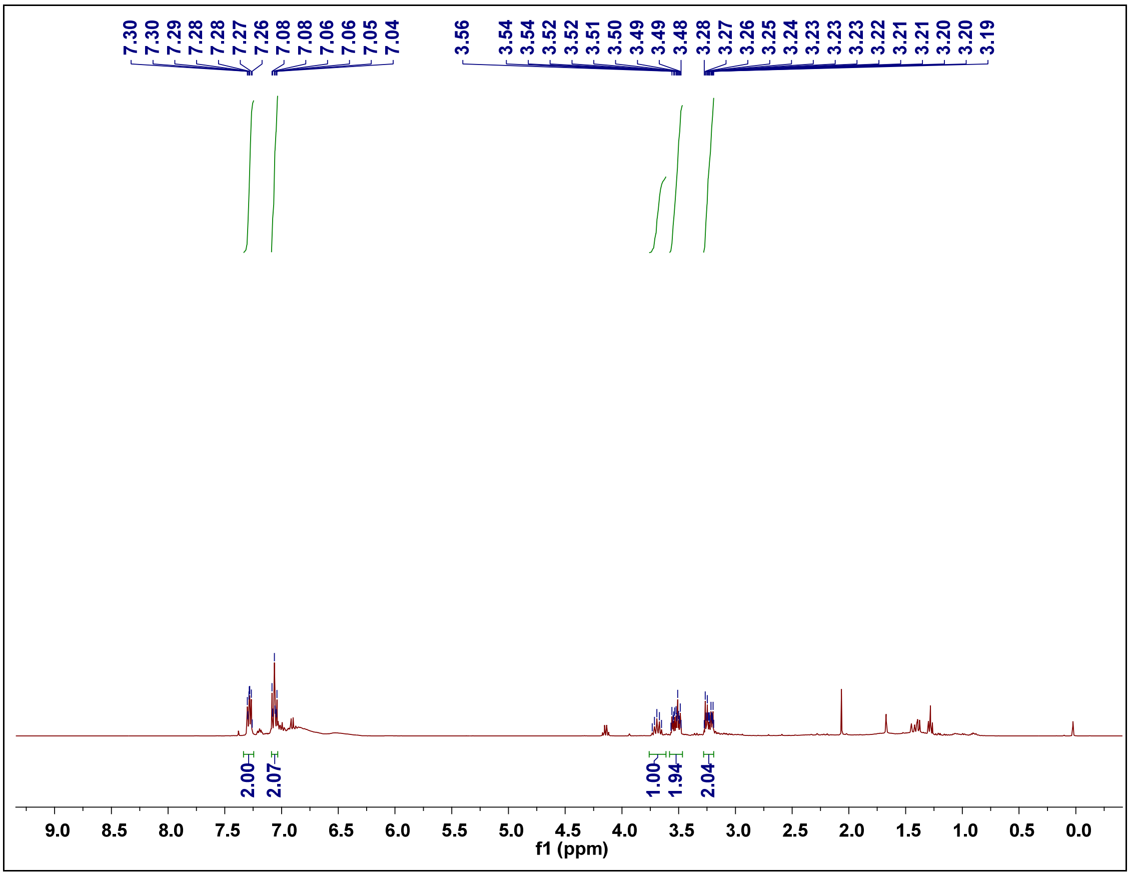

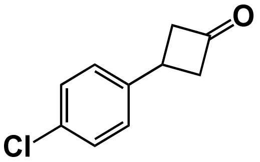

3-(4-chlorophenyl)cyclobutanone (3a): 1-chloro-4-vinylbenzene (1.60 g, 11.6 mmol) was used as raw material, yielding colorless oil. Yield: 1.69 g (65%). ^{1}H NMR (400 MHz, $CDCl_3$, ppm): δ 7.35-7.32 (m, 2H), 7.25 (d, J = 8.8 Hz, 2H), 3.72-3.64 (m, 1H), 3.56-3.48 (m, 2H), 3.27-3.18 (m, 2H).

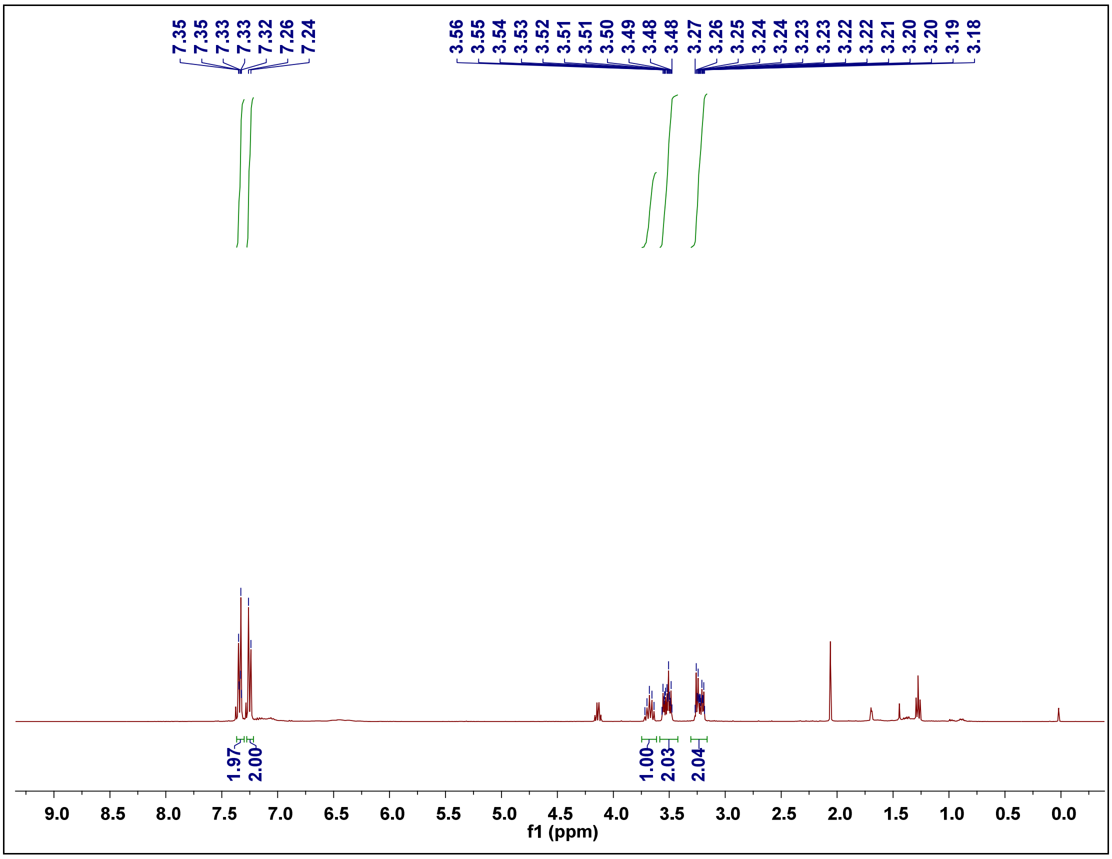

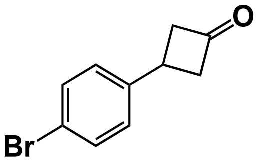

3-(4-bromophenyl)cyclobutanone (4a): 1-bromo-4-vinylbenzene (2.12 g, 11.6 mmol) was used as raw material, yielding colorless oil. Yield: 1.81 g (69%). ^{1}H NMR (400 MHz, $CDCl_3$, ppm): δ 7.50-7.47(m, 2H), 7.21-7.17 (m, 2H), 3.70-3.62 (m, 1H), 3.55-3.47 (m, 2H), 3.26-3.18 (m, 2H).

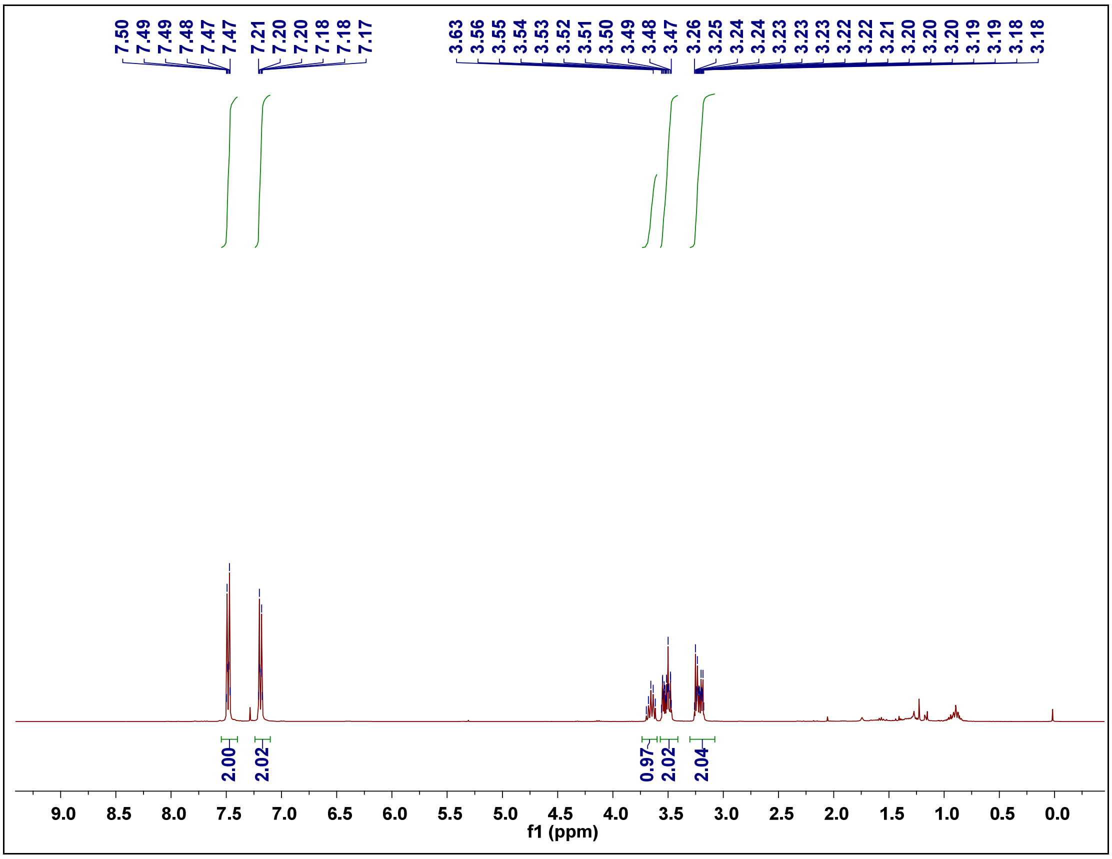

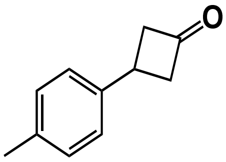

3-*p*-tolylcyclobutanone (5a): 1-methyl-4-vinylbenzene (1.37 g, 11.6 mmol) was used as raw material, yielding colorless oil. Yield: 0.66 g (36%). ^{1}H NMR (400 MHz, $CDCl_3$, ppm): δ 7.25-7.19 (m, 4H), 3.72-3.64 (m, 1H), 3.53-3.47 (m, 2H), 3.29-3.22 (m, 2H), 2.39 (s, 3H).

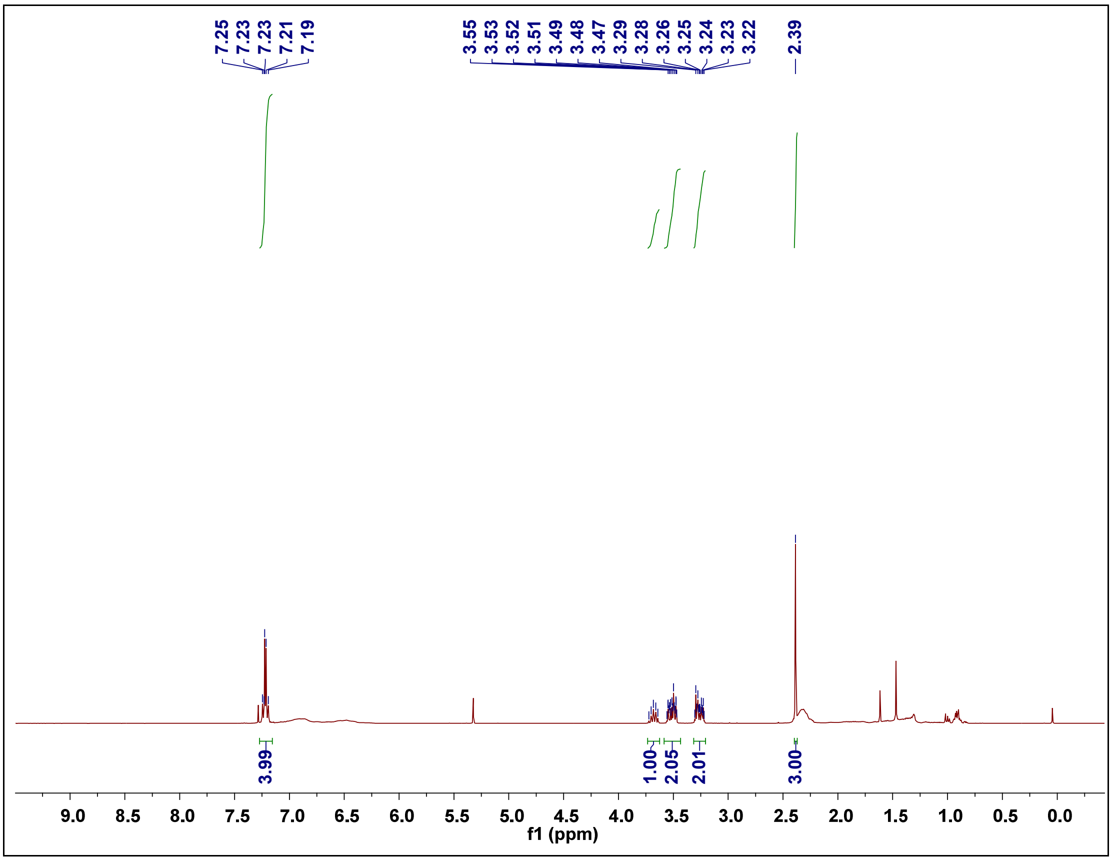

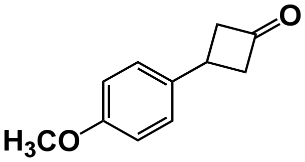

3-(4-methoxybenzyl)cyclobutanone (6a): 1-allyl-4-methoxybenzene (1.72 g, 11.6 mmol) was used as raw material, yielding colorless oil. Yield: 1.20 g (63%). ^{1}H NMR (400 MHz, $CDCl_3$, ppm): δ 7.23-7.20 (m, 2H), 6.90-6.87 (m, 2H), 3.80 (s, 3H), 3.67-3.59 (m, 2H), 3.50-3.42 (m, 2H), 3.24-3.15 (m, 2H).

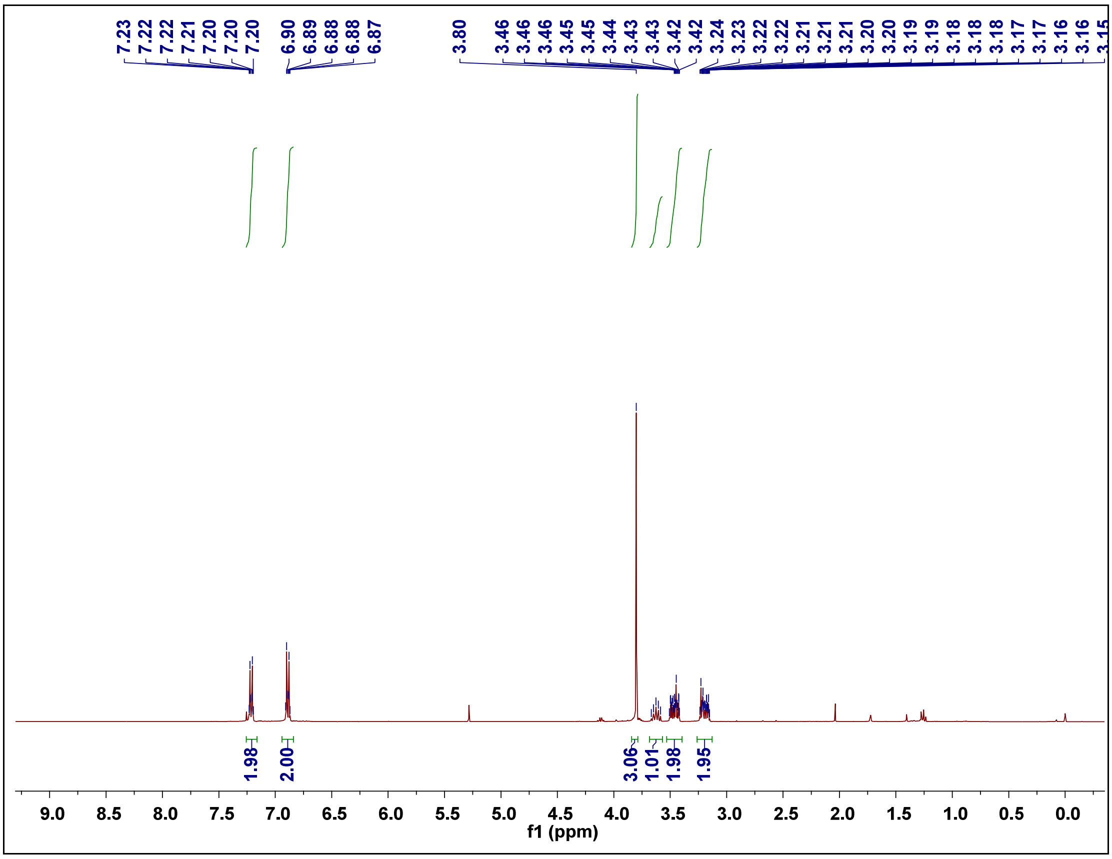

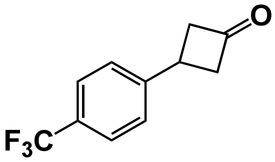

3-(4-(trifluoromethyl)phenyl)cyclobutanone (7a): 1-trifluoromethyl-4-vinylbenzene (1.99 g, 11.6 mmol) was used as raw material, yielding colorless oil. Yield: 1.63 g (66%). ^{1}H NMR (400 MHz, $CDCl_3$, ppm): δ 7.62 (d, J = 8.4 Hz, 2H), 7.42 (d, J = 8.4 Hz, 2H), 3.79-3.71 (m, 1H), 3.59-3.51 (m, 2H), 3.31-3.22 (m, 2H).

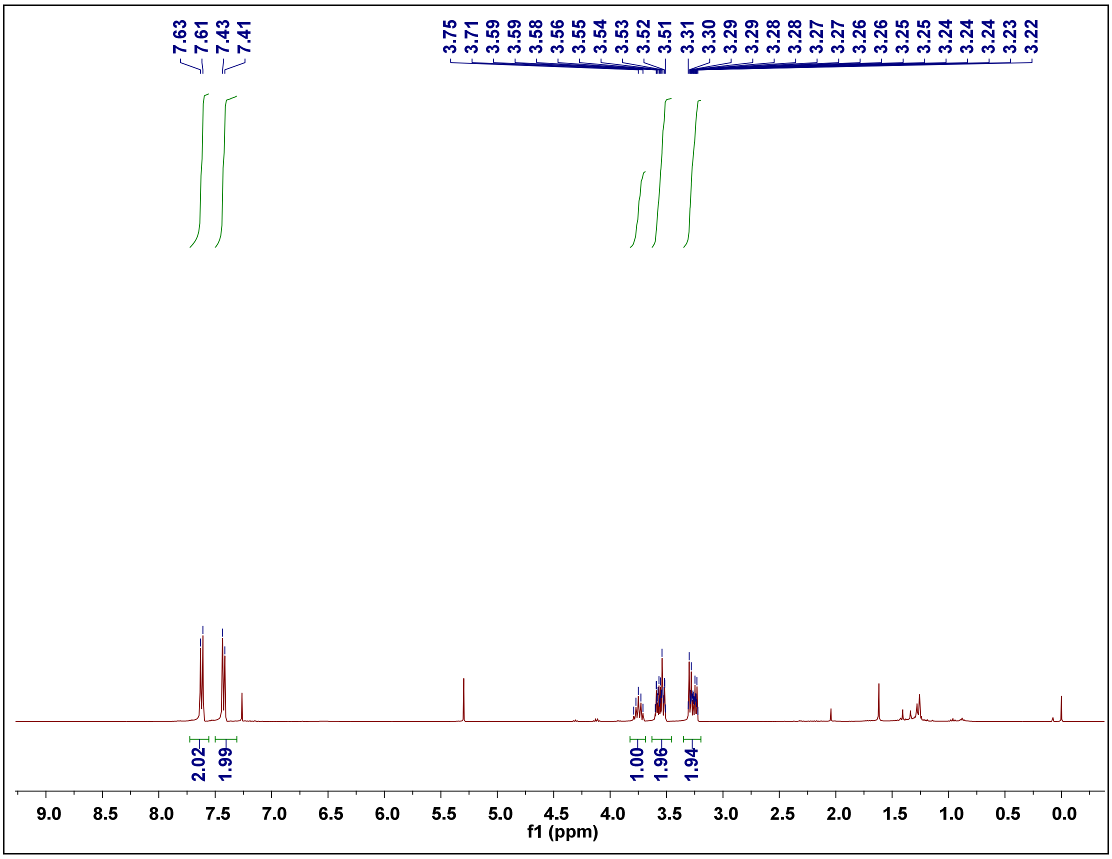

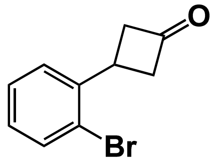

3-(2-bromophenyl)cyclobutanone (8a): 1-bromo-2-vinylbenzene (2.12 g, 11.6 mmol) was used as raw material, yielding colorless oil. Yield: 1.78 g (68%). ^{1}H NMR (400 MHz, $CDCl_3$, ppm): δ 7.64-7.62 (m, 1H), 7.39-7.34 (m, 2H), 7.19-7.14 (m, 1H), 4.02-3.93 (m, 1H), 3.57-3.51 (m, 2H), 3.29-3.20 (m, 2H).

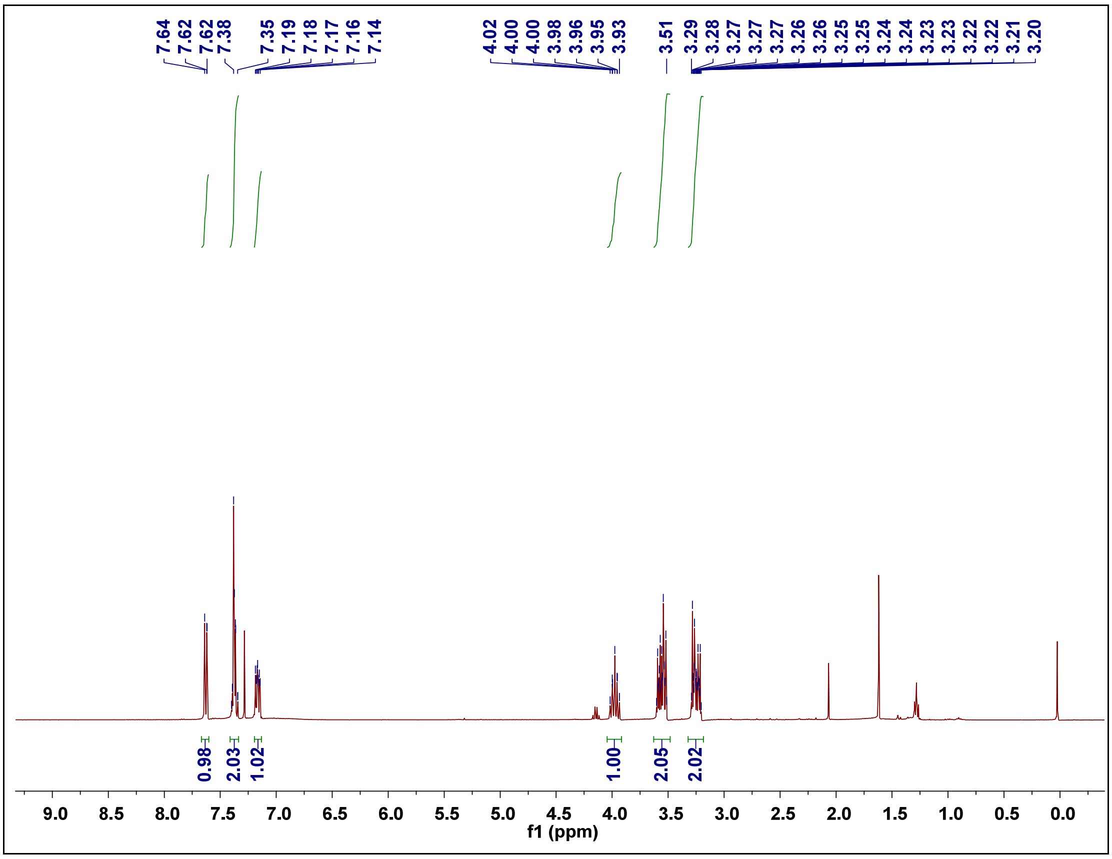

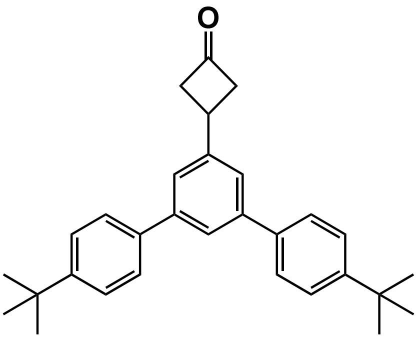

3-(4,4''-di-tert-butyl-[1,1':3',1''-terphenyl]-5'-yl)cyclobutanone (9a): 4,4''-di-tert-butyl-5'-vinyl-1,1':3',1''-terphenyl (4.27 g, 11.6 mmol) was used as raw material, yielding a white solid. Yield: 2.47 g (52%). ^{1}H NMR (400 MHz, $CDCl_3$, ppm): δ 7.68 (s, J = 1.6 Hz, 1H), 7.57 (d, J = 8.0 Hz, 4H), 7.49 (d, J = 8.0 Hz, 4H), 7.46 (s, 2H), 3.83-3.77 (m, 1H), 3.58-3.53 (m, 2H), 3.39-3.34 (m, 2H) , 7.46 (s, 18H).

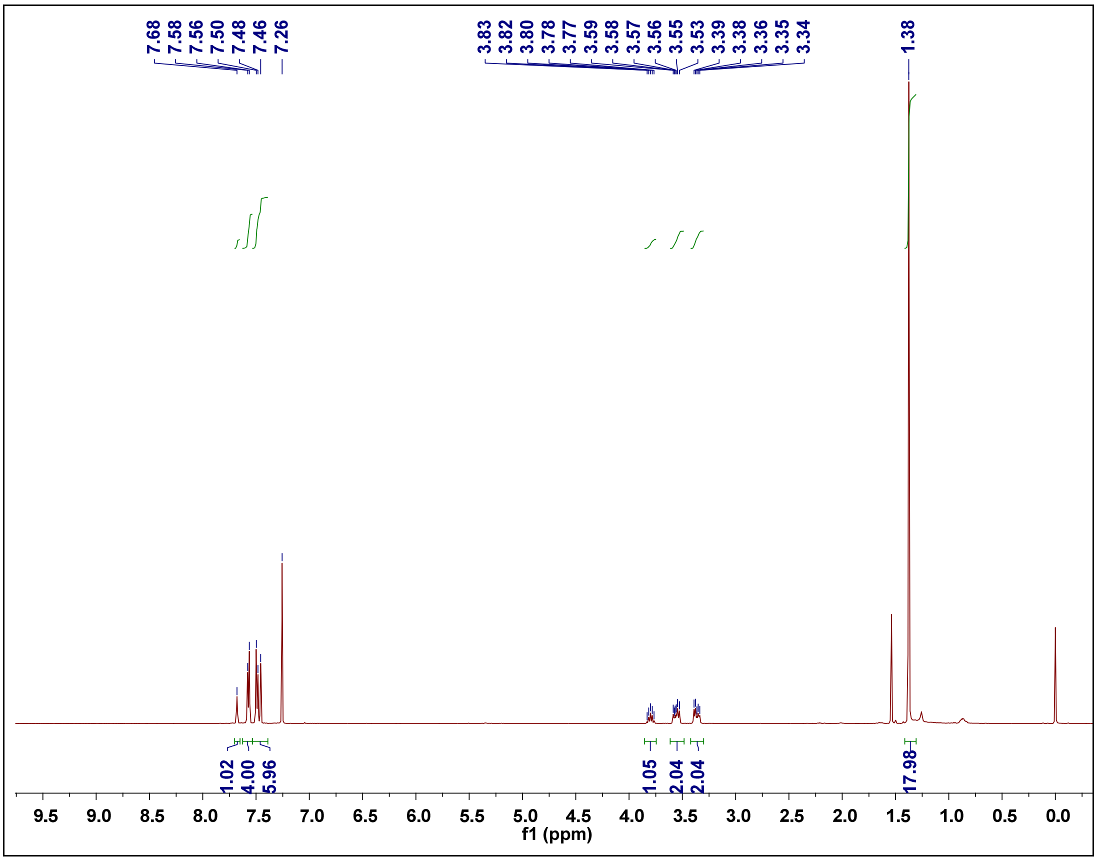

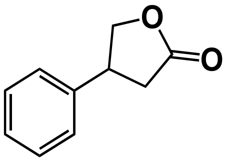

4-Phenyldihydrofuran-2(3H)-one (1b): (400 MHz, $CDCl_3$, ppm): δ 7.39-7.35 (m, 2H), 7.32-7.29 (m, 1H), 7.25-7.22 (m, 2H), 4.68-4.64 (m, 1H), 4.27-4.24 (m, 1H), 3.83-3.74 (m, 1H), 2.91 (dd, J = 17.5, 8.7 Hz, 1H), 2.68 (dd, J = 17.5, 8.7 Hz, 1H).

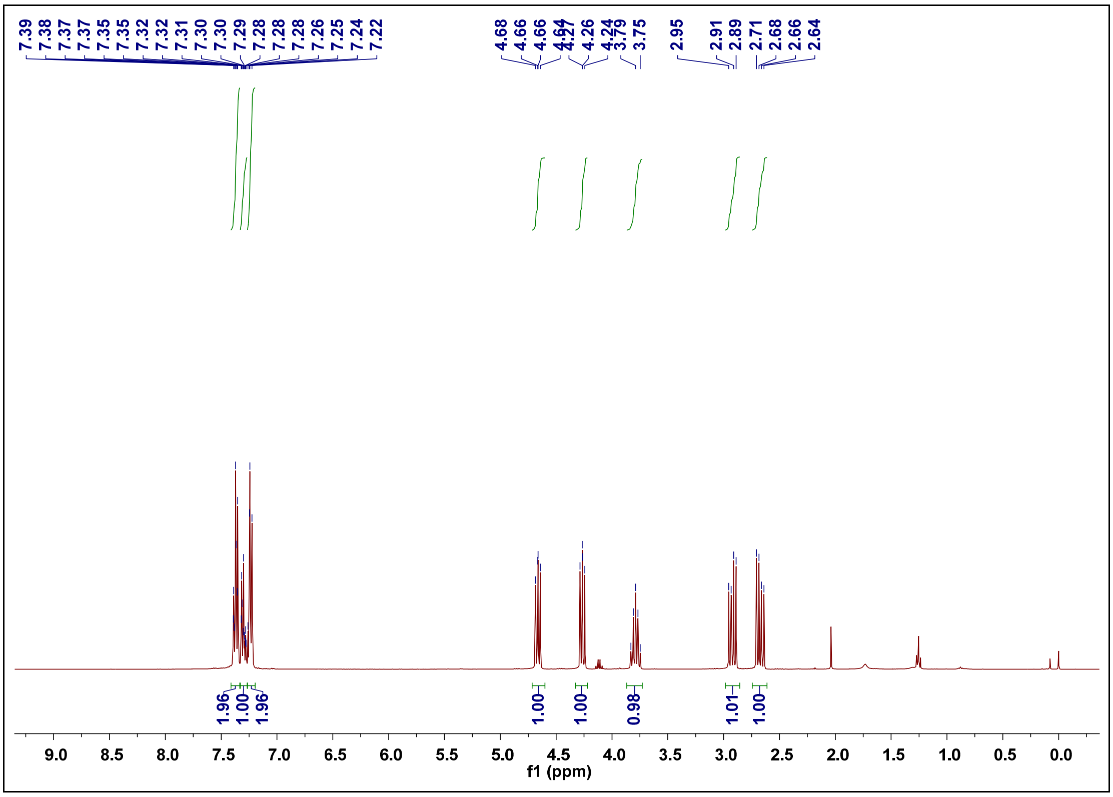

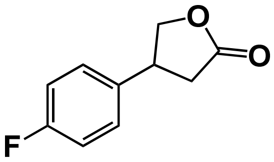

4-(4-Fluorophenyl)dihydrofuran-2(3H)-one (2b): (400 MHz, $CDCl_3$, ppm): δ 7.22-7.18 (m, 2H), 7.07-7.02 (m, 2H), 4.67-4.62 (m, 1H), 4.24-4.20 (m, 1H), 3.81-3.73 (m, 1H), 2.91 (dd, J = 17.6, 8.8 Hz, 1H), 2.62 (dd, J = 17.6, 8.8 Hz, 1H).

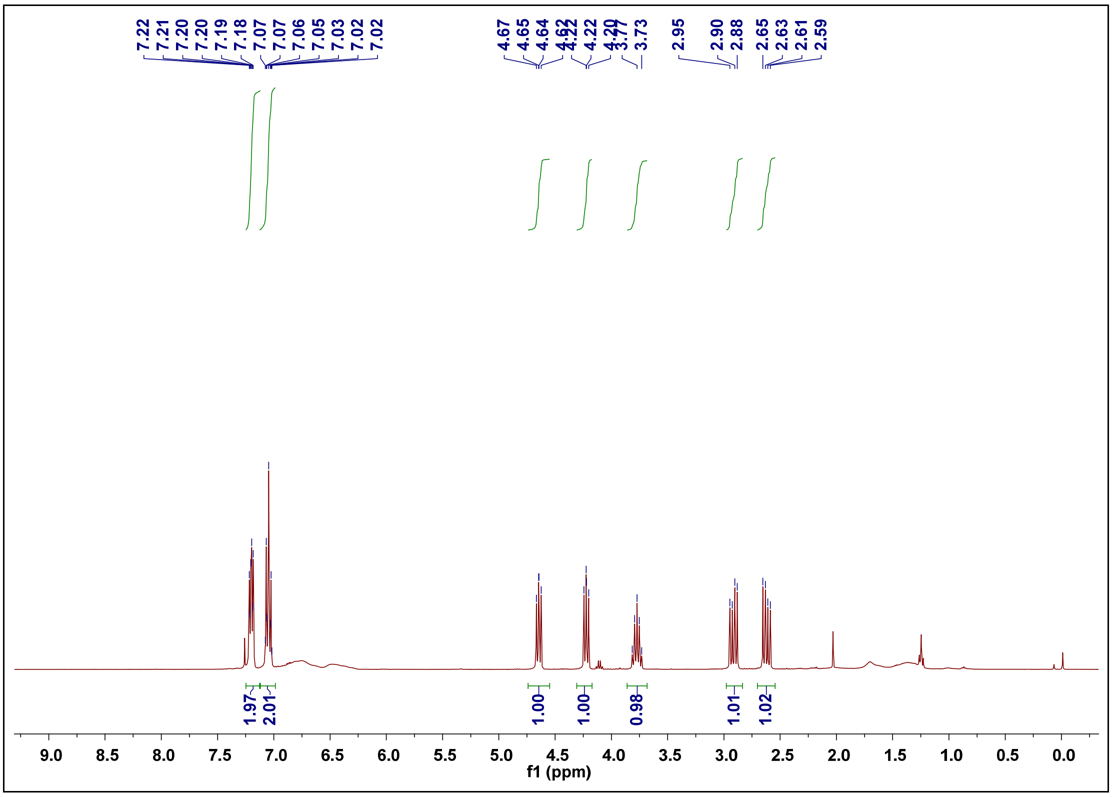

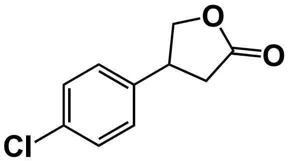

4-(4-Chlorophenyl)dihydrofuran-2(3H)-one (3b): (400 MHz, $CDCl_3$, ppm): *δ* 7.38-7.36 (m, 2H), 7.19 (d, *J* = 8.4 Hz, 2H), 4.70-4.66 (m, 1H), 4.28-4.24 (m, 1H), 3.83-3.75 (m, 1H), 2.95 (dd, *J* = 17.4, 8.7 Hz, 1H), 2.65 (dd, *J* = 17.4, 8.7 Hz, 1H).

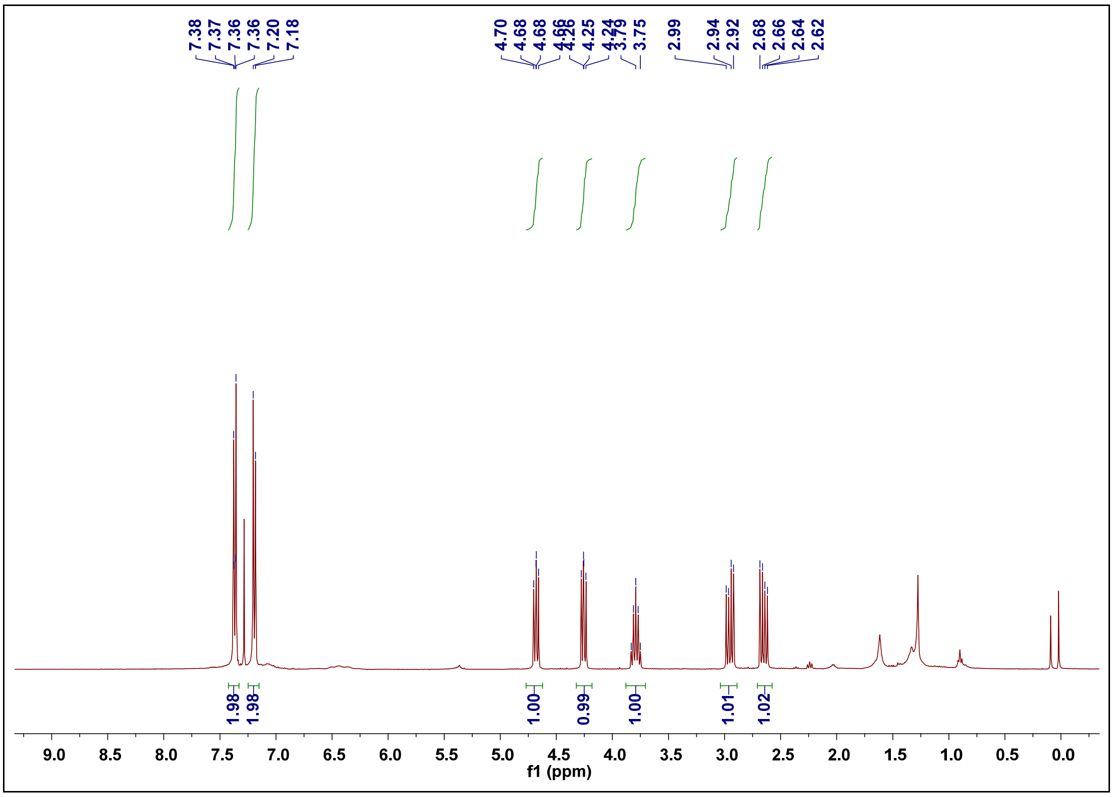

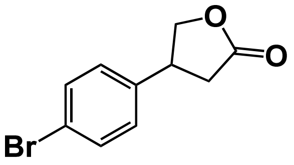

4-(4-Bromophenyl)dihydrofuran-2(3H)-one (4b): (400 MHz, $CDCl_3$, ppm): δ 7.49-7.46 (m, 2H), 7.12-7.09 (m, 2H), 4.66-4.62 (m, 1H), 4.23-4.19 (m, 1H), 3.79-3.70 (m, 1H), 2.91 (dd, J = 17.6, 8.8 Hz, 1H), 2.61 (dd, J = 17.6, 8.8 Hz, 1H).

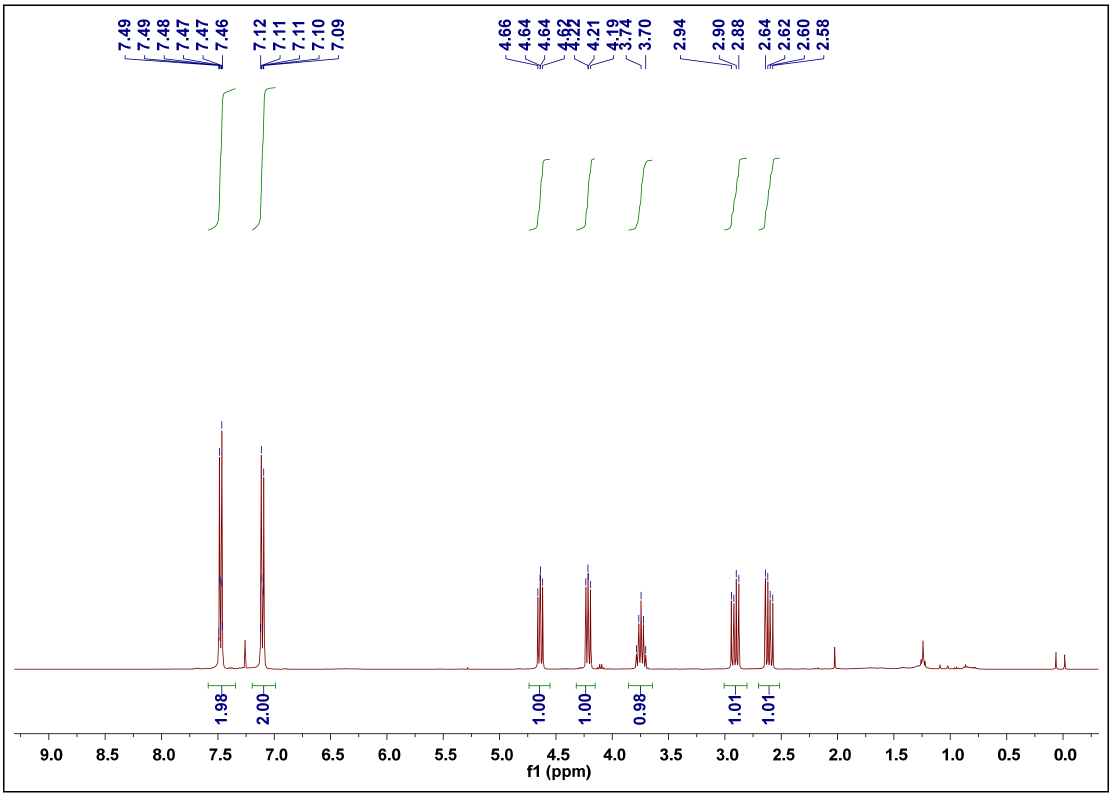

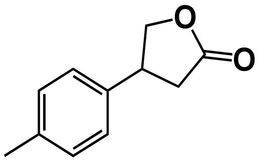

4-p-Tolyldihydrofuran-2(3H)-one (5b): (400 MHz, $CDCl_3$, ppm): δ 7.18 (d, J = 8.0 Hz, 2H), 7.12 (d, J = 8.0 Hz, 2H), 4.67-4.62 (m, 1H), 4.26-4.22 (t, J = 8.0 Hz, 1H), 3.79-3.71 (m, 1H), 2.90 (dd, J = 17.6, 8.8 Hz, 1H), 2.65 (dd, J = 17.6, 8.8 Hz, 1H), 2.35 (s, 1H).

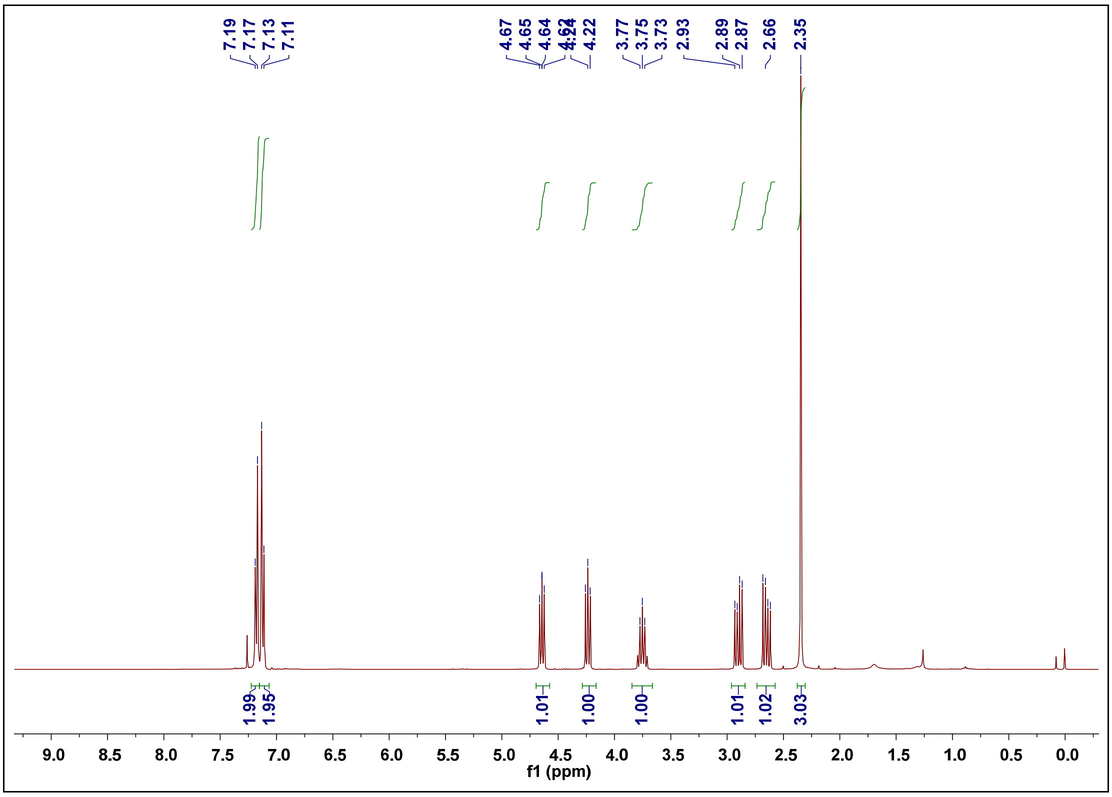

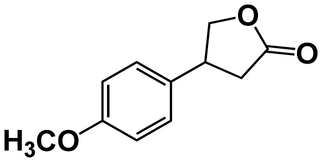

4-(4-Methoxyphenyl)dihydrofuran-2(3H)-one (6b): (400 MHz, $CDCl_3$, ppm): δ 7.19-7.15 (m, 2H), 6.93-6.89 (m, 2H), 4.66-4.62 (m, 1H), 4.25-4.21 (t, *J* = 8.0 Hz, 1H), 3.81 (s, 1H), 3.79-3.71 (m, 1H), 2.90 (dd, *J* = 17.4, 8.7 Hz, 1H), 2.64 (dd, *J* = 17.4, 8.7 Hz, 1H).

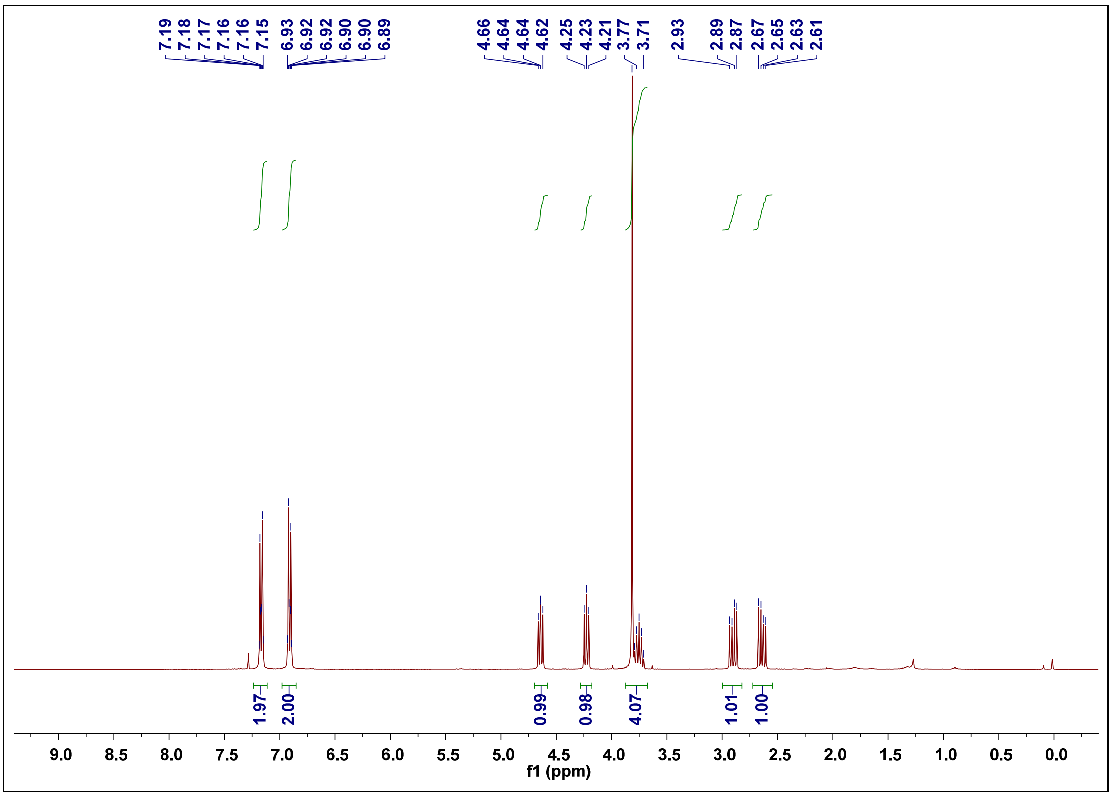

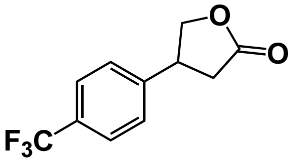

4-(4-(Trifluoromethyl)phenyl)dihydrofuran-2(3H)-one (7b): (400 MHz, $CDCl_3$, ppm): δ 7.63 (d, J = 8.0 Hz, 2H), 7.36 (d, J = 8.0 Hz, 2H), 4.71-4.67 (m, 1H), 4.30-4.26 (m, 1H), 3.90-3.82 (m, 1H), 2.96 (dd, J = 17.6, 8.8 Hz, 1H), 2.66 (dd, J = 17.6, 8.8 Hz, 1H).

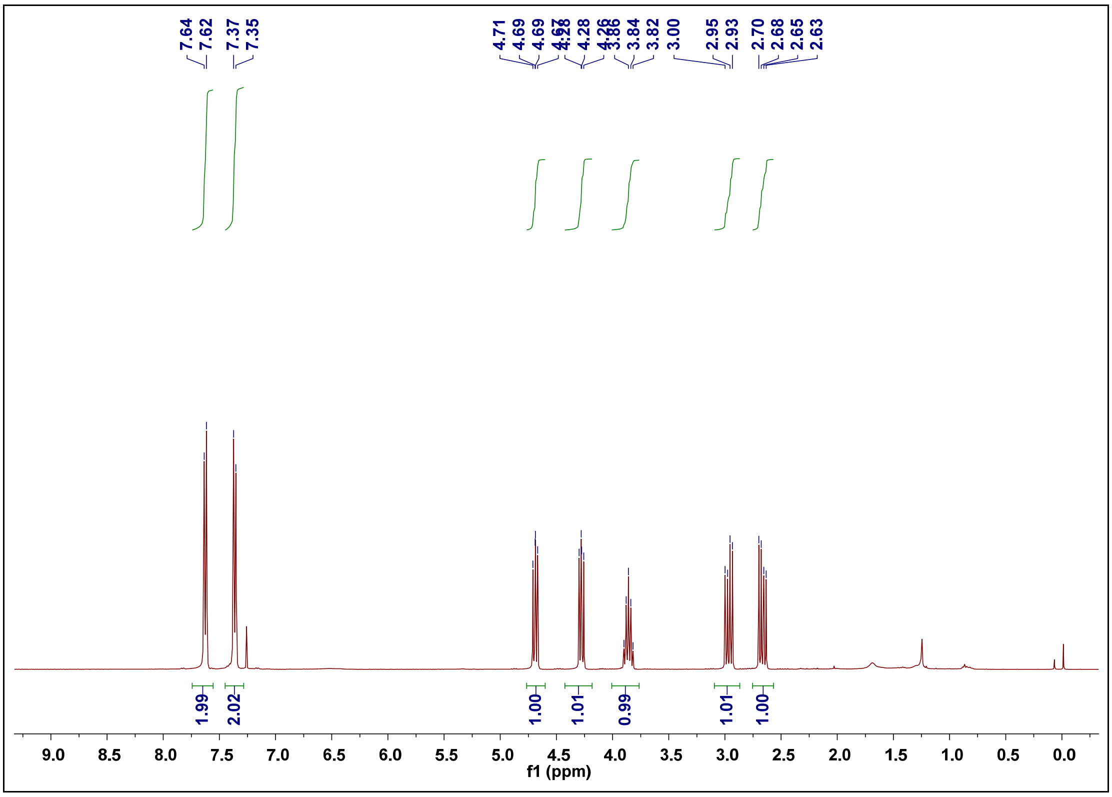

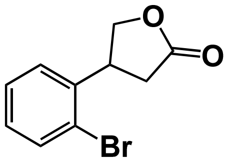

4-(2-Bromophenyl)dihydrofuran-2(3H)-one (8b): (400 MHz, $CDCl_3$, ppm): δ 7.63 (dd, J = 8.0, 1.2 Hz, 1H), 7.37-7.30 (m, 1H), 7.30-4.26 (m, 1H), 7.36 (td, J = 5.6, 1.6 Hz, 1H), 4.73-4.69 (m, 1H), 4.33-4.29 (m, 1H), 4.27-4.19 (m, 1H), 2.98 (dd, J = 17.6, 8.8 Hz, 1H), 2.67 (dd, J = 17.6, 8.8 Hz, 1H).

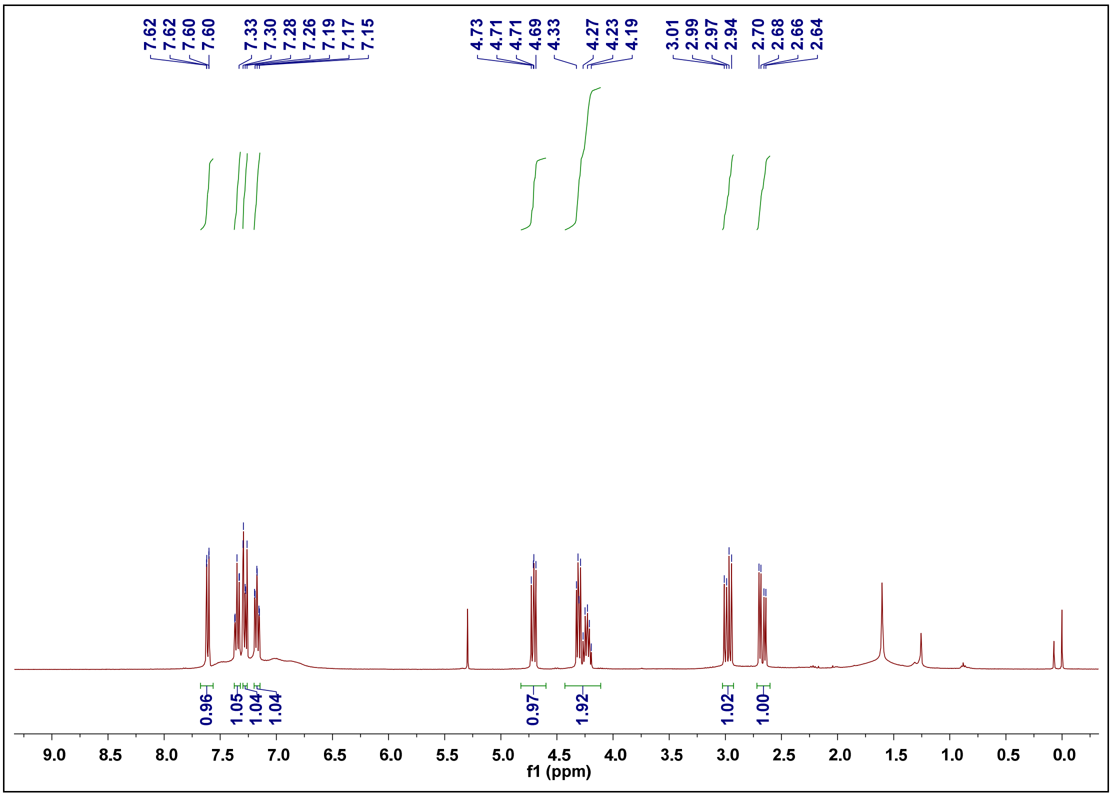

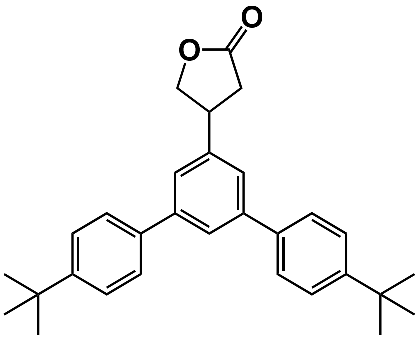

4-(4,4''-di-tert-butyl-[1,1':3',1''-terphenyl]-5'-yl)dihydrofuran-2(3H)-one (9b): (400 MHz, $CDCl_3$, ppm): δ 7.76 (t, J = 1.6, 1H), 7.58-7.56 (m, 4H), 7.52-7.50 (m, 4H), 7.39 (d, J = 1.6, 2H), 4.78-4.74 (m, 1H), 4.42-4.38 (m, 1H), 3.97-3.89 (m, 1H), 3.02 (dd, J = 17.6, 8.8 Hz, 1H), 2.81 (dd, J = 17.6, 8.8 Hz, 1H), 1.40 (s, 18H).

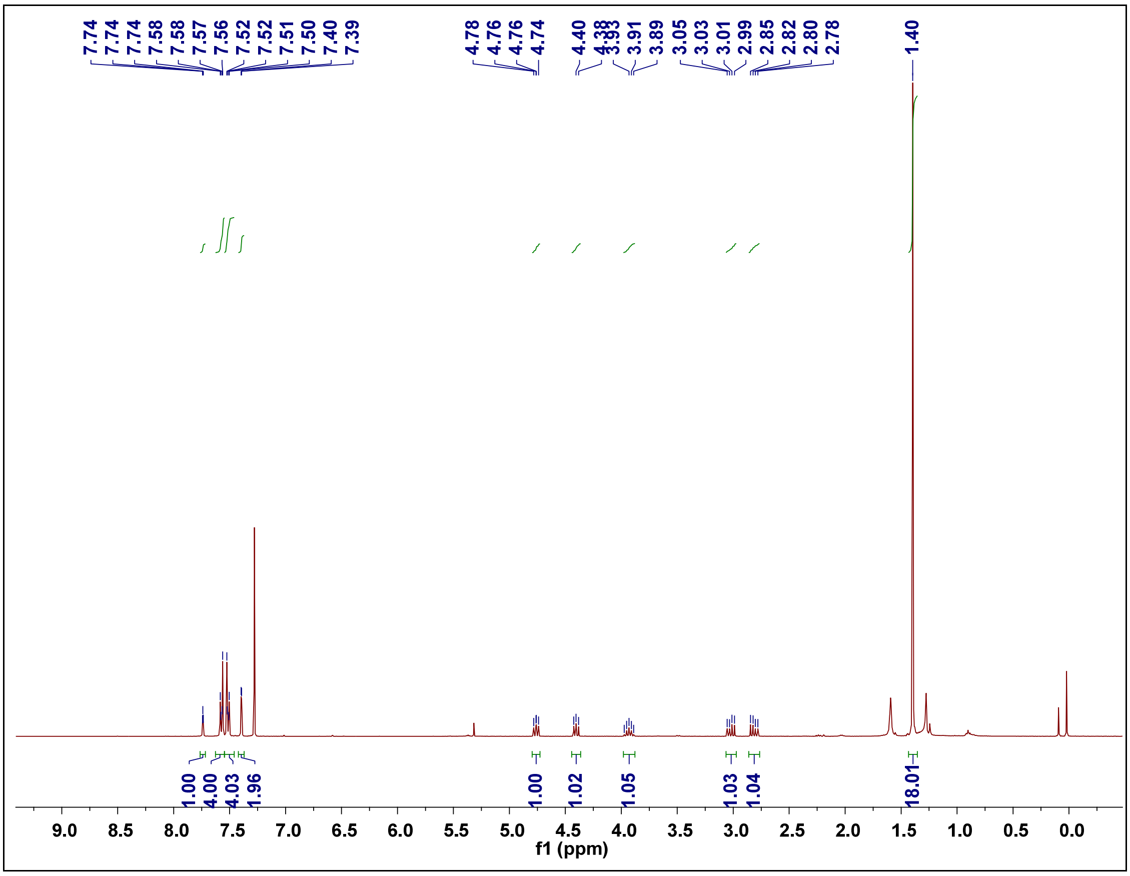

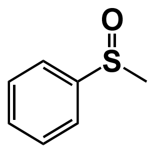

(Methylsulfinyl)benzene (1d): (400 MHz, $CDCl_3$, ppm): *δ* 7.65-7.63 (m, 2H), 7.54-7.47 (m, 3H), 2.71 (s, 3H).

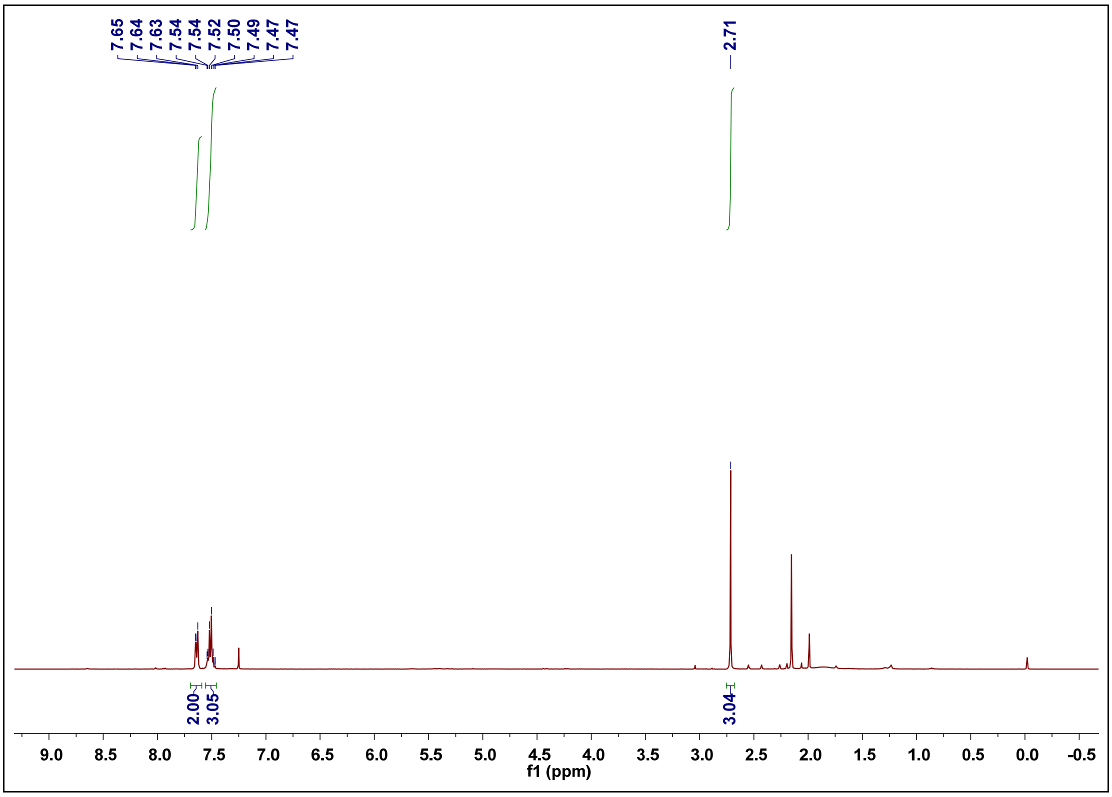

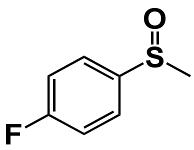

1-Fluoro-4-(methylsulfinyl)benzene (2d): (400 MHz, $CDCl_3$, ppm): δ 7.66-7.63 (m, 2H), 7.22 (t, J = 17.6, 8.4 Hz, 2H), 2.71 (s, 3H).

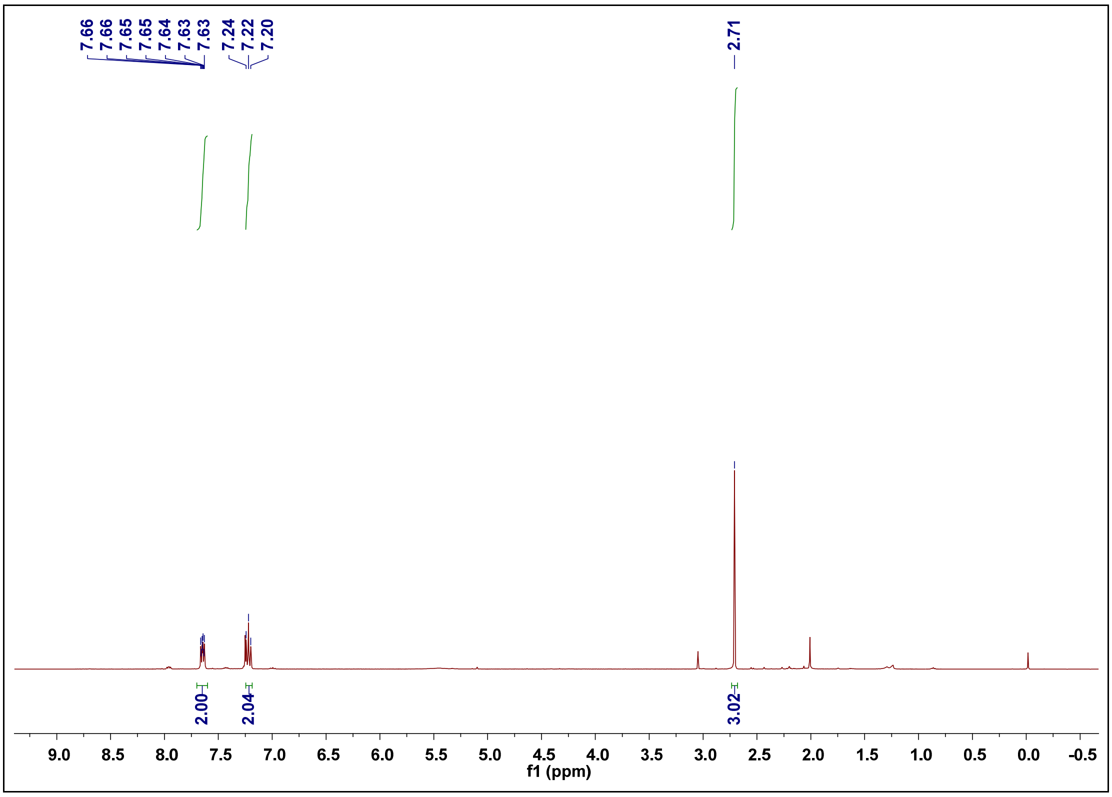

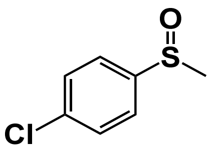

1-Chloro-4-(methylsulfinyl)benzene (3d): (400 MHz, $CDCl_3$, ppm): δ 7.62 (d, J = 8.4, 2H), 7.54 (d, J = 8.4 Hz, 2H), 2.74 (s, 3H).

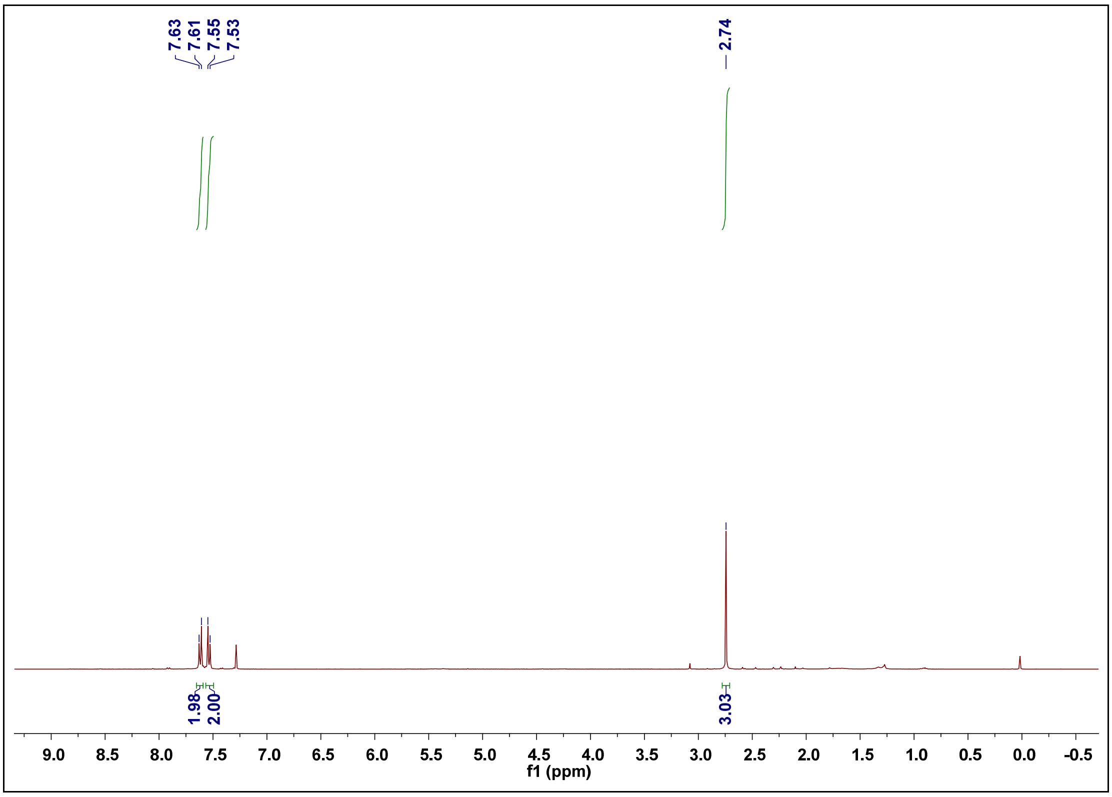

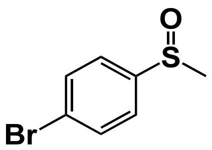

1-Bromo-4-(methylsulfinyl)benzene (4d): (400 MHz, $CDCl_3$, ppm): δ 7.66 (d, J = 8.4, 2H), 7.51 (d, J = 8.4 Hz, 2H), 2.71 (s, 3H).

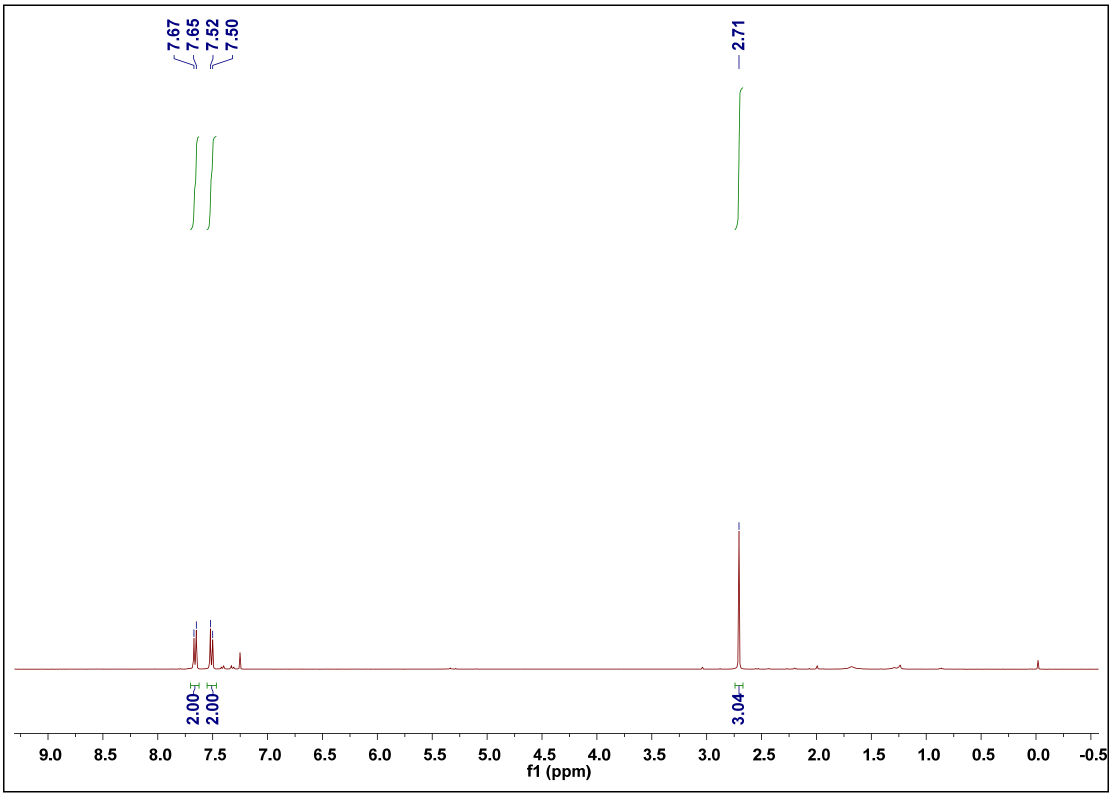

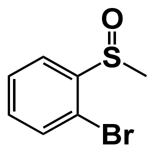

1-Bromo-2-(methylsulfinyl)benzene (5d): (400 MHz, $CDCl_3$, ppm): *δ* 7.94 (dd, *J* = 7.6, 1.2 Hz, 1H), 7.59-7.55 (m, 2H), 7.39-7.34 (m, 1H), 2.81 (s, 3H).

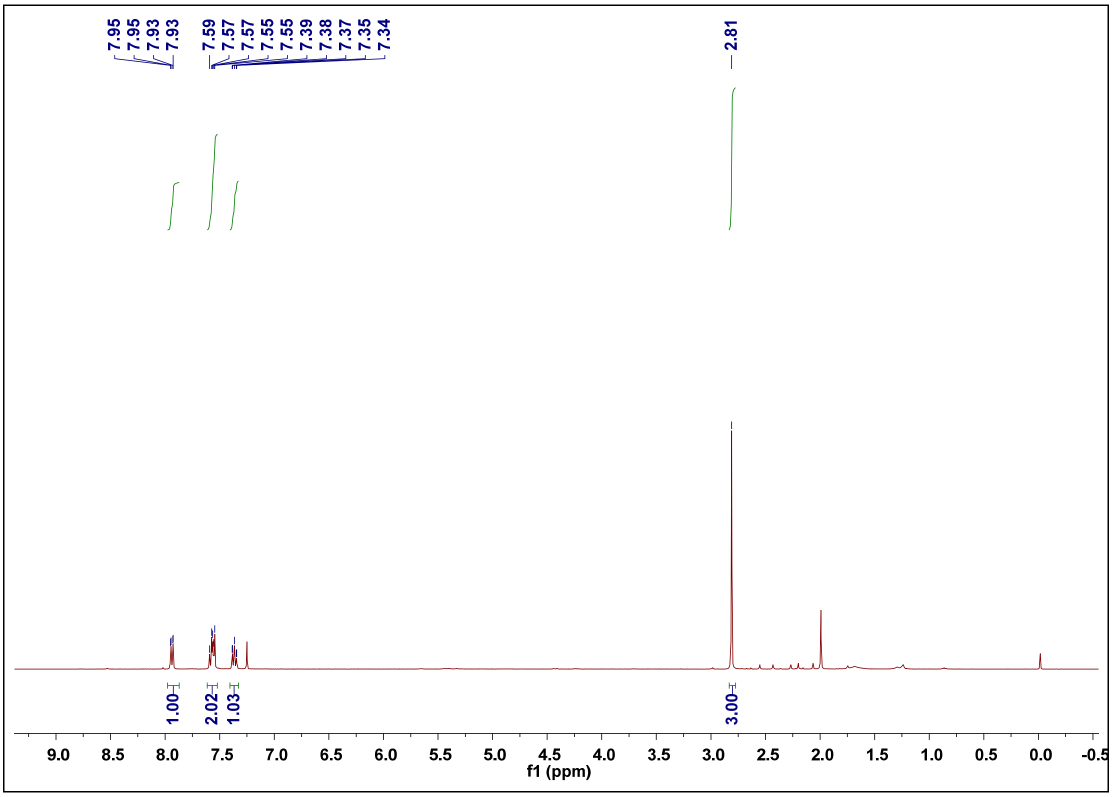

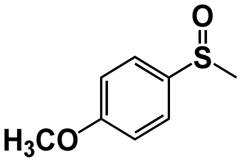

1-Methoxy-4-(methylsulfinyl)benzene (6d): (400 MHz, $CDCl_3$, ppm): δ 7.58 (d, J = 8.8, 2H), 7.02 (d, J = 8.8 Hz, 2H), 3.84 (s, 3H), 2.69 (s, 3H).

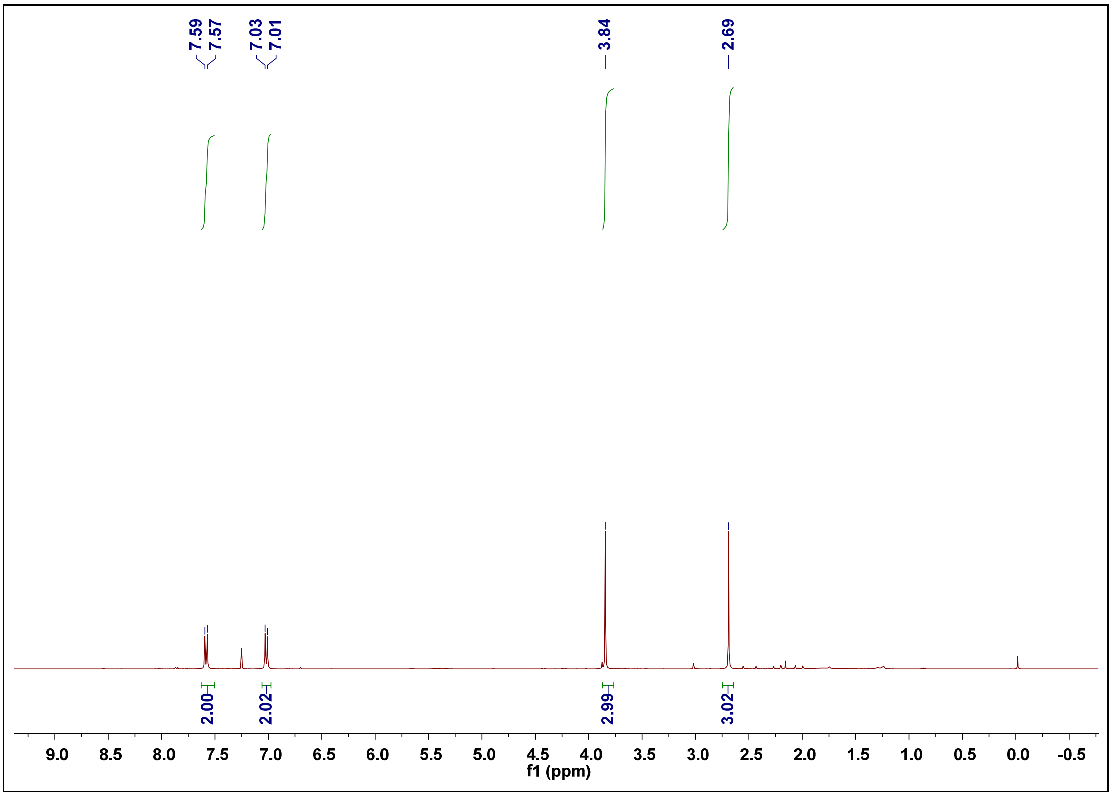

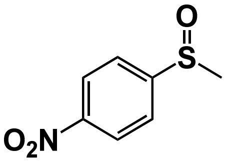

1-Nitro-4-(methylsulfinyl)benzene (7d): (400 MHz, $CDCl_3$, ppm): δ 8.40 (d, J = 7.2, 2H), 7.85 (d, J = 7.2 Hz, 2H), 2.80 (s, 3H).

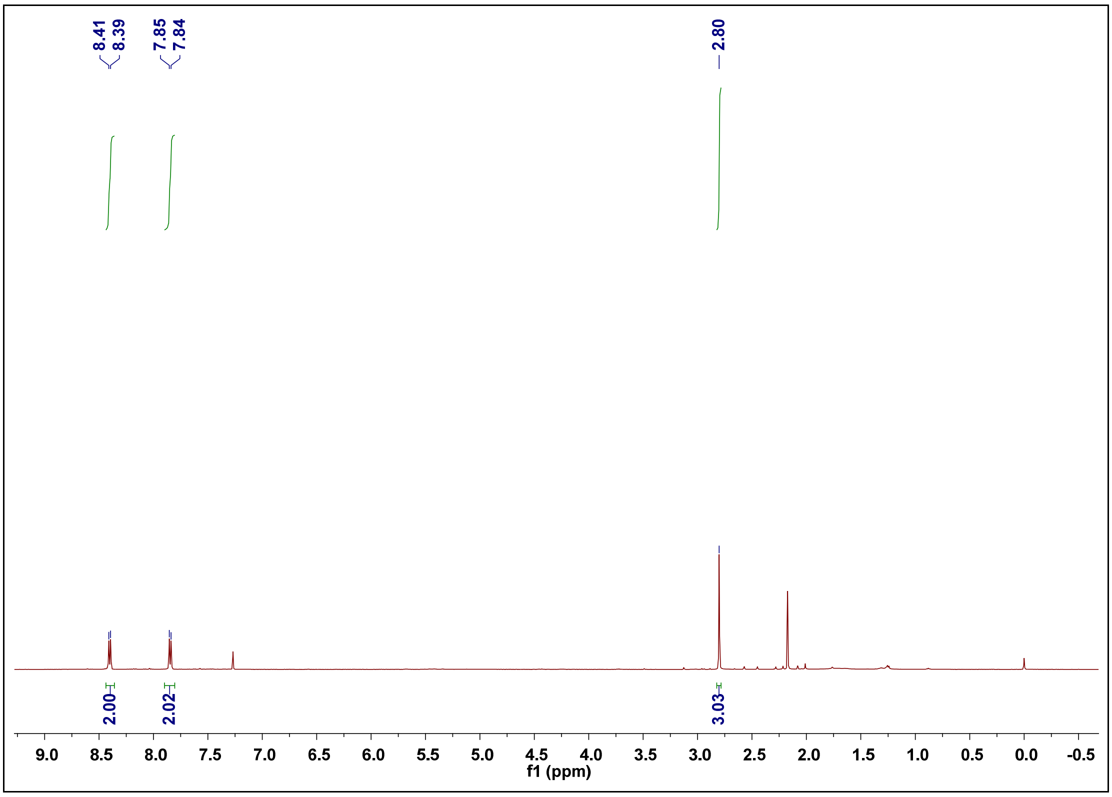

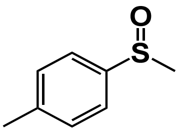

1-Methyl-4-(methylsulfinyl)benzene (8d): (400 MHz, $CDCl_3$, ppm): δ 7.56 (d, J = 8.4, 2H), 7.35 (d, J = 8.4 Hz, 2H), 2.72 (s, 3H), 2.44 (s, 3H).

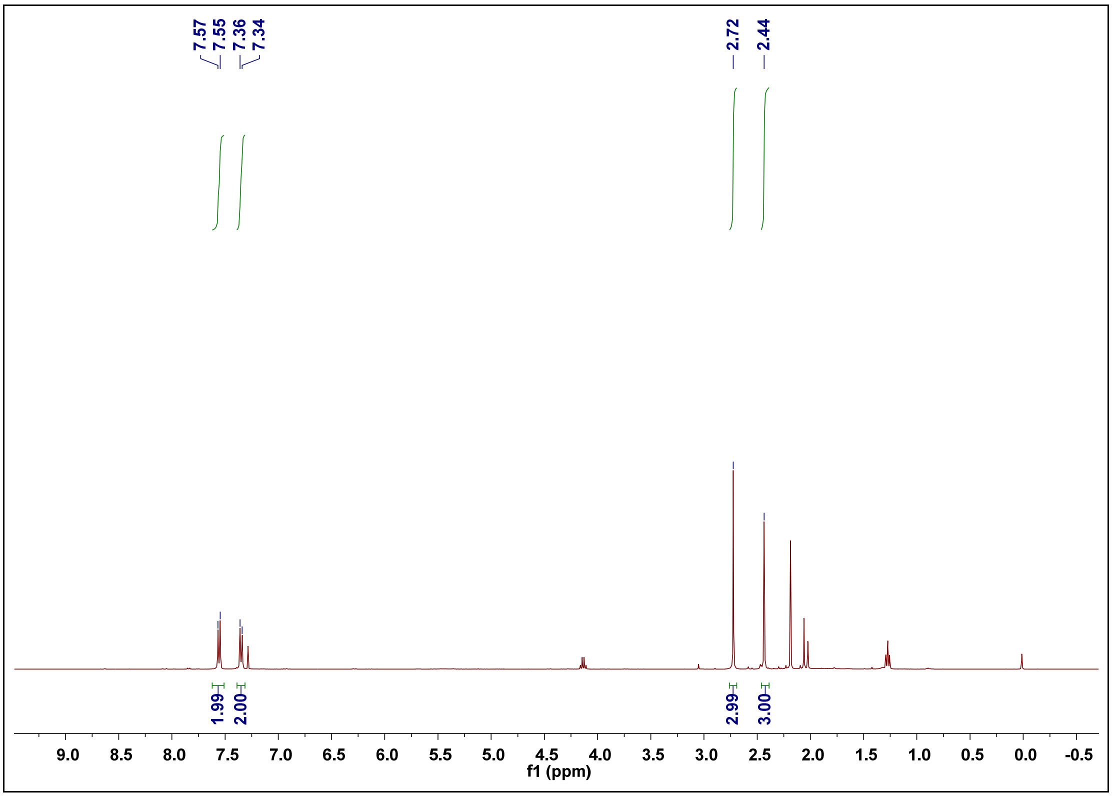

Supplementary References

1. Mai, A., Valente, S., Meade, S., Carafa, V., Tardugno, M., Nebbioso, A., Galmozzi, A., Mitro, N., Fabiani, E. D., Altucci, L. & Kazantsev, A. Study of 1,4-dihydropyridine structural scaffold: discovery of novel sirtuin activators and inhibitors. *J. Med. Chem.* **52**, 5496−5504 (2009).

2. Ranford, J. D., Vittal, J. J. & Wang, Y. M. Dicopper(II) complexes of the antitumor analogues acylbis(salicylaldehyde hydrazones) and crystal structures of monomeric [Cu_2(1,3-propanedioyl bis(salicylaldehyde hydrazone)) $(H_2O)_2$]·$(ClO_4)_2$·$3H_2O$ and polymeric [{Cu_2(1,6-hexanedioyl bis(salicylaldehyde hydrazone))$(C_2H_5OH)_2$}$_m$] $(ClO_4)_{2m}$·m(C_2H_5OH). *Inorg. Chem.* **37**, 1226−1231 (1998).

3. Liu, T., Zou, L., Feng, D., Chen, Y., Fordham, S., Wang, X., Liu, Y. & Zhou, H. Stepwise synthesis of robust metal−organic frameworks via postsynthetic metathesis and oxidation of metal nodes in a single-crystal to single-crystal transformation. *J. Am. Chem. Soc.* **136**, 7813−7816 (2014).

4. Bezencon, O., Heidmann, B., Siegrist, R., Stamm, S., Richard, S., Pozzi, D., Corminboeuf, O., Roch, C., Kessler, M., Ertel, E. A., Reymond, I., Pfeifer, T., Kanter, R., Toeroek-Schafroth, M., Moccia, L. G., Mawet, J., Moon, R., Rey, M., Capeleto, B. & Fournier, E. Discovery of a potent, selective T-type calcium channel blocker as a drug candidate for the treatment of generalized epilepsies. *J. Med. Chem.* **60**, 9769−9789 (2017).

5. SMART, Data collection software (version 5.629) (Bruker AXS Inc., Madison, WI, 2003).

6. SAINT, Data reduction software (version 6.45) (Bruker AXS Inc.; Madison, WI, 2003).

7. Sheldrick, G. M. SHELXTL97, Program for Crystal Structure Solution (University of Göttingen: Göttingen, Germany, 1997).

8. Spek, A. L. Single-crystal structure validation with the program PLATON. *J. Appl. Cryst.* **36***,* 7−13 (2003).
